# An anti-FAP-scFv-functionalized exosome-carrying hydrogel delivers *SKI* mRNA to fibrotic nucleus pulposus cells to alleviate intervertebral disc degeneration by regulating FOXO3

**DOI:** 10.7150/thno.107776

**Published:** 2025-03-03

**Authors:** Yueyang Li, Yu Zhai, Tianling Wang, Siya Wang, Yu Tian, Zhiqun Bian, Yang Zhang, Chao Zhang, Minghan Liu, Changqing Li

**Affiliations:** 1 Department of Orthopedics, Xinqiao Hospital, Third Military Medical University (Army Medical University), 400038, Chongqing, China.; 2 Chongqing Municipal Health Commission Key Laboratory of Precision Orthopaedic, 400038, Chongqing, China.; 3 State Key Laboratory of Trauma and Chemical Poisoning, 400038, Chongqing, China.; 4 College of Bioengineering, Chongqing University, 400044, Chongqing, China.; 5 Department Of Wound Repair and Rehabilitation Medicine, Center of Bone Metabolism and Repair, State Key Laboratory of Trauma, Burns and Combined Injury, Trauma Center, Research Institute of Surgery, Daping Hospital, Army Medical University, Chongqing, China.

**Keywords:** intervertebral disc degeneration, nucleus pulposus fibrosis, *SKI* gene, functionalized exosomes, pH-responsive hydrogel

## Abstract

**Rationale:** Nucleus pulposus (NP) fibrosis is a contributing factor to intervertebral disc degeneration (IVDD), which lacks an effective treatment. This study focuses on elucidating the role and mechanisms of the TGF-β signaling repressor *SKI* in NP fibrosis and exploring its therapeutic potential.

**Methods:** Single-cell RNA sequencing (scRNA-seq) was used to investigate fibrotic nucleus pulposus cell (NPC) subsets and assess TGF-β signaling activation. Two recombinant plasmids encoding single-chain variable fragments (scFvs) targeting the fibrotic NPC marker FAP and *SKI* mRNA were co-transfected into HEK-293T cells to yield functionalized exosomes (EX^ski+scFv^). The addition of EX^ski+scFv^ to a gelatin/oxidized sodium alginate hydrogel produced a pH-responsive exosome/hydrogel system named Gel@EX^ski+scFv^. The therapeutic effects and underlying mechanism of Gel@EX^ski+scFv^ were evaluated by RNA sequencing, molecular docking and coimmunoprecipitation.

**Results:** A fibrotic NPC subset was characterized by elevated FAP and diminished *SKI* expression, along with activation of the TGF-β signaling pathway. *SKI* overexpression reduced fibrosis in TGF-β-treated NPCs. EX^ski+scFv^ successfully delivered *SKI* mRNA to FAP-expressing fibrotic NPCs. Gel@EX^ski+scFv^ possessed good mechanical properties, degradability, injectability, and biocompatibility. Gel@EX^ski+scFv^ effectively alleviated NP fibrosis and IVDD in rats. RNA sequencing, molecular docking and coimmunoprecipitation revealed that *SKI* could interact with FOXO3 to suppress the TGF-β signaling pathway.

**Conclusion:** This study elucidates the mechanism by which *SKI*-mediated TGF-β signaling inhibition alleviates NP fibrosis. The development of the Gel@EX^ski+scFv^ system for the targeted delivery of *SKI* mRNA offers a promising therapeutic strategy to alleviate NP fibrosis and IVDD in the future.

## Introduction

Intervertebral disc degeneration (IVDD) is a common orthopedic condition that affects individuals of all ages worldwide. IVDD can impair quality of life, reduce work efficacy, and impose a major socioeconomic burden 1. Current clinical treatments for IVDD are mainly conservative or involve surgery; however, these treatments cannot reverse IVDD progression 2. Fibrosis in nucleus pulposus (NP) tissue is a characteristic manifestation of IVDD and a key exacerbating factor 3. NP fibrosis manifests mainly as extracellular matrix (ECM) remodeling in nucleus pulposus cells (NPCs), decreased NP elasticity, irreversible and severe tissue remodeling and abnormal collagen matrix deposition, which eventually lead to structural and functional impairment of the NP tissue 4, 5. Although inhibiting NP fibrosis has shown potential in alleviating IVDD, to date, few clinically effective antifibrotic therapies have been developed to treat NP fibrosis 6.

The activation of the TGF-β signaling pathway is a classic inducer of many fibrosis-related diseases 7, 8. The TGF-β signaling pathway plays crucial roles in physiological and pathological processes within intervertebral discs (IVDs) 9. Under physiological conditions, TGF-β is involved in ECM synthesis and secretion 10, 11. However, numerous studies have shown that excessive activation of TGF-β signaling may lead to the occurrence of IVDD 12, 13, and previous studies have shown that IVDD is accompanied by a substantial increase in TGF-β expression in IVDs 14, 15. We hypothesize that inhibiting TGF-β signaling may alleviate NP fibrosis and contribute to IVDD treatment. However, the literature suggests that inhibiting TGF-β signaling may be associated with severe off-target effects 16, 17, which may also adversely affect the nonfibrotic parts of the tissue. Therefore, targeted inhibition of the TGF-β signaling pathway in fibrotic NPCs is needed to accurately regulate NP fibrosis.

The proto-oncogene* SKI* (Cellular Sloan Kettering Institute) is a transcriptional regulator and a classic repressor of TGF-β signaling 18.* SKI* has been shown to inhibit pulmonary 19, skin 20, kidney 21, and myocardial 22 fibrosis. These data suggest that *SKI* may have potential for the treatment of NP fibrosis. However, most of the recently reported *SKI* therapy methods for fibrosis rely on virus-mediated overexpression, which could result in the induction of inflammation and toxic reactions that may alter its therapeutic effect 23. In addition, virus-mediated overexpression may lead to viral integration into the genome 24. Moreover, repeated drug administration is needed to achieve stable and sustained gene overexpression, which may cause undesirable damage to IVDs.

Exosomes (Exos) are lipid nanovesicles secreted by cells that range in size from 50- to 200 nm 25. In recent years, Exos have been widely used as drug nanodelivery systems. Exos can carry various biomolecules, including proteins, nucleic acids and metabolites, and effectively deliver these molecules through interactions with recipient cells 26. Notably, exosomal delivery of mRNAs is a promising therapeutic strategy with potential for gene therapy applications 27. mRNA can be directly used for protein synthesis via intracellular RNA translation and has the advantage of instantaneous expression. However, mRNA delivery needs to overcome the extracellular barrier to avoid degradation by RNA hydrolase in the extracellular serum 28. Importantly, the lipid composition of Exos can increase their stability, thus helping to avoid mRNA degradation by the extracellular barrier and reducing recognition for mononuclear phagocytosis. mRNA can be loaded into Exos secreted by living cells through active preloading, passive preloading, and postloading methods 29. On this basis, modifying Exos with single-chain variable fragments (scFvs), oligonucleotides, peptides, small-molecule compounds or other materials can endow Exos with the ability to target specific cells or specific cell surface proteins 30, 31. Therefore, the use of Exos as delivery carriers may allow the targeted delivery of *SKI* mRNA to fibrotic NPCs, facilitating precise NP fibrosis treatment.

In this study, we identified fibrotic NPC subsets in degenerative IVDs via single-cell RNA sequencing (scRNA-seq). We found that TGF-β signaling was activated and that the *SKI* expression level was decreased in fibrotic NPCs. Moreover, the expression of a specific fibrotic marker, the membrane protein fibroblast activation protein-α (FAP), was increased in fibrotic NPCs. Therefore, after confirming the antifibrotic effect of *SKI* mRNA *in vitro*, we used the active preloading method to construct two recombinant plasmids for cotransfection into HEK-293T cells to produce EX^ski+scFv^, which can achieve targeted delivery of loaded *SKI* mRNA to fibrotic NPCs. Furthermore, encapsulating EX^ski+scFv^ in a pH-responsive gelatin-oxidized sodium alginate (OSA) hydrogel yielded Gel@EX^ski+scFv^, which inhibited fibrosis and alleviated IVDD in a TGF-β-induced NP fibrosis rat model. RNA sequencing, molecular docking and coimmunoprecipitation experiments demonstrated that *SKI* inhibited TGF-β signaling by the binding of the SKI protein to FOXO3. This study provides a novel strategy for the future realization of clinically accurate and efficient therapeutic strategies for treating NP fibrosis, facilitating the treatment of IVDD.

## Results

### The scRNA-seq atlas revealed fibrosis-related changes and TGF-β signaling activation in IVDD

**Figure 1:**
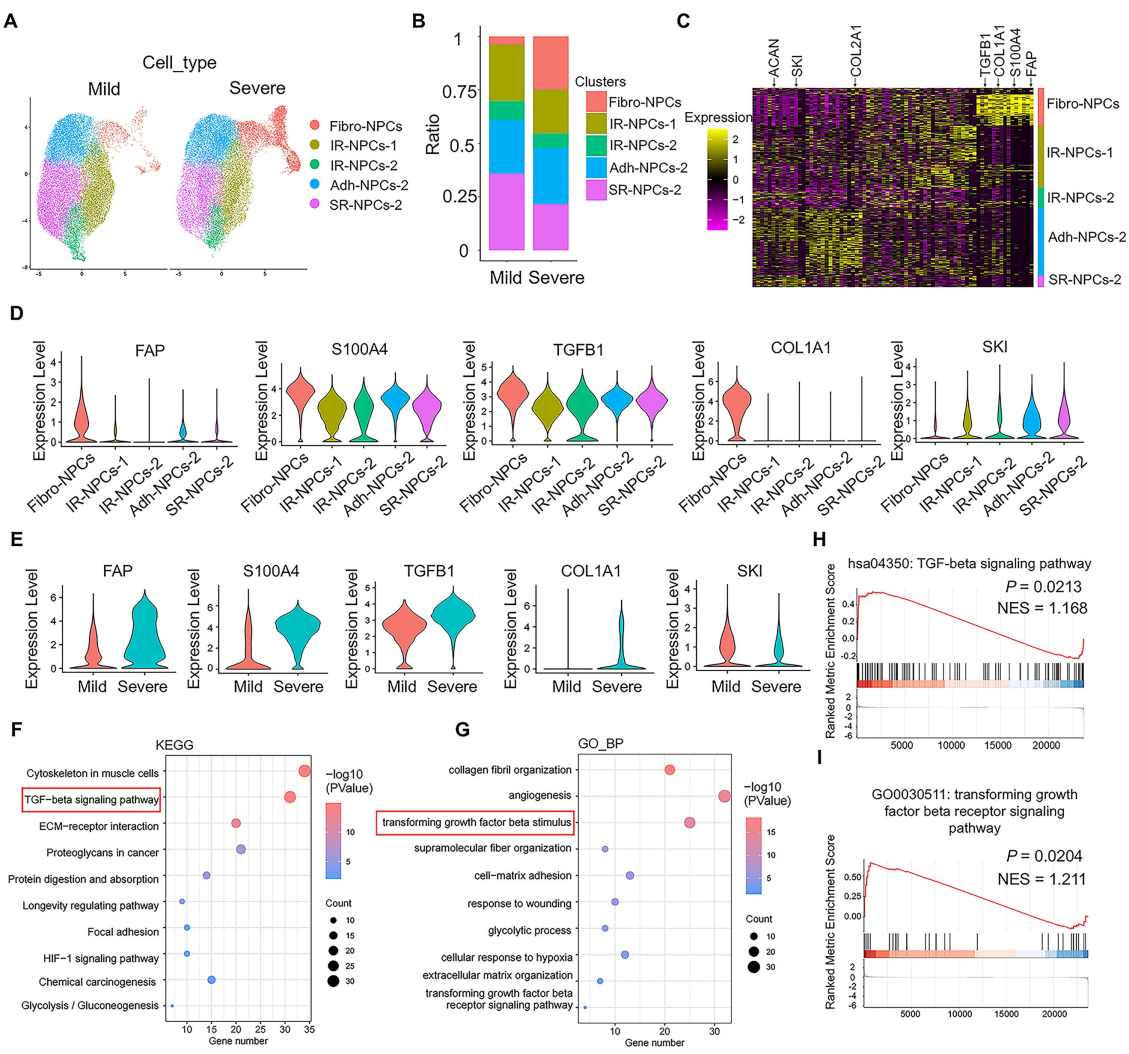
**Identification of fibrotic NPC subsets and TGF-β signaling activation by scRNA-seq. (A)** Schematic UMAP of each subset of NPCs in the mild and severe degenerative groups with a resolution of 0.5. **(B)** Proportion of each subset of NPCs among the total number of cells in the mild and severe degenerative groups. **(C)** Heatmap of differentially expressed genes in each subpopulation of NPCs. **(D)** Comparison of the DEGs between the fibrotic NPC subset and the other subsets. The thresholds were log2FC > 0.25; P Adj < 0.05; and min.pct > 0.1. **(E)** Comparison of the DEGs in the fibrotic NPC subset between the mild and severe degenerative groups; the thresholds were log2FC > 0.25; P < 0.05; and min.pct > 0.1. **(F-G)** NPC GO term and KEGG pathway enrichment analyses were performed using DAVID online. **(H-I)** GSEA of the NPCs was performed using the clusterProfiler package in R (version: 4.4.4).

scRNA-seq analysis was performed on the mild and severe degenerative NP tissue dataset (GSE244889). The total cells in the NP tissue were divided into 12 subsets **(Figure S1A)**, and the NPCs were divided into 5 subsets: fibrotic NPCs (fibro-NPCs), integral regulatory NPCs (IR-NPCs-1 and IR-NPCs-2), adhesive NPCs (Adh-NPCs), and stress-responsive NPCs (SR-NPCs) **(Figure S1B)**. The proportion of the fibro-NPC subset was significantly greater in severely degenerative NP tissue than in mildly degenerative NP tissue **(Figure 1A-B)**. The heatmap shows that *COL2A1* and *ACAN*, which represent NPCs, were expressed mainly in the IR-NPCs-1, IR-NPCs-2, Adh-NPC, and SR-NPC subsets, whereas *FAP*, *S100A4*, and *COL1A1*, which represent fibrotic cells, were expressed mainly in fibro-NPCs **(Figure 1C)**. Moreover, a positive regulator of TGF-β signaling, *TGFB1*, was upregulated in these subsets, whereas a negative regulator of TGF-β signaling, *SKI*, was downregulated **(Figure 1C-D)**. In addition, the expression of *FAP*, *S100A4*, *COL1A1*, and *TGFB1* in the fibro-NPC subset tended to be upregulated in severely degenerative NP tissue compared with mildly degenerative NP tissue, whereas the expression of *SKI* in the fibro-NPC subset tended to be downregulated **(Figure 1E)**. Kyoto Encyclopedia of Genes and Genomes (KEGG) and Gene Ontology (GO) analyses revealed that the terms “TGF-beta signaling pathway” and “cellular response to transforming growth factor β stimulus” were enriched **(Figure 1F-G)**. Several ECM remodeling-related terms were also enriched, as indicated by GO cellular component (CC) and molecular function (MF) analyses **(Figure S1C-D)**. Moreover, gene set enrichment analysis (GSEA) revealed a trend toward TGF-β signaling activation **(Figure 1H-I)**. These results suggest that IVDD is accompanied by fibrotic-like changes in NPCs and increased activation of TGF-β signaling.

### Activating TGF-β signaling and reducing *SKI* levels contributed to NP fibrosis

**Figure 2:**
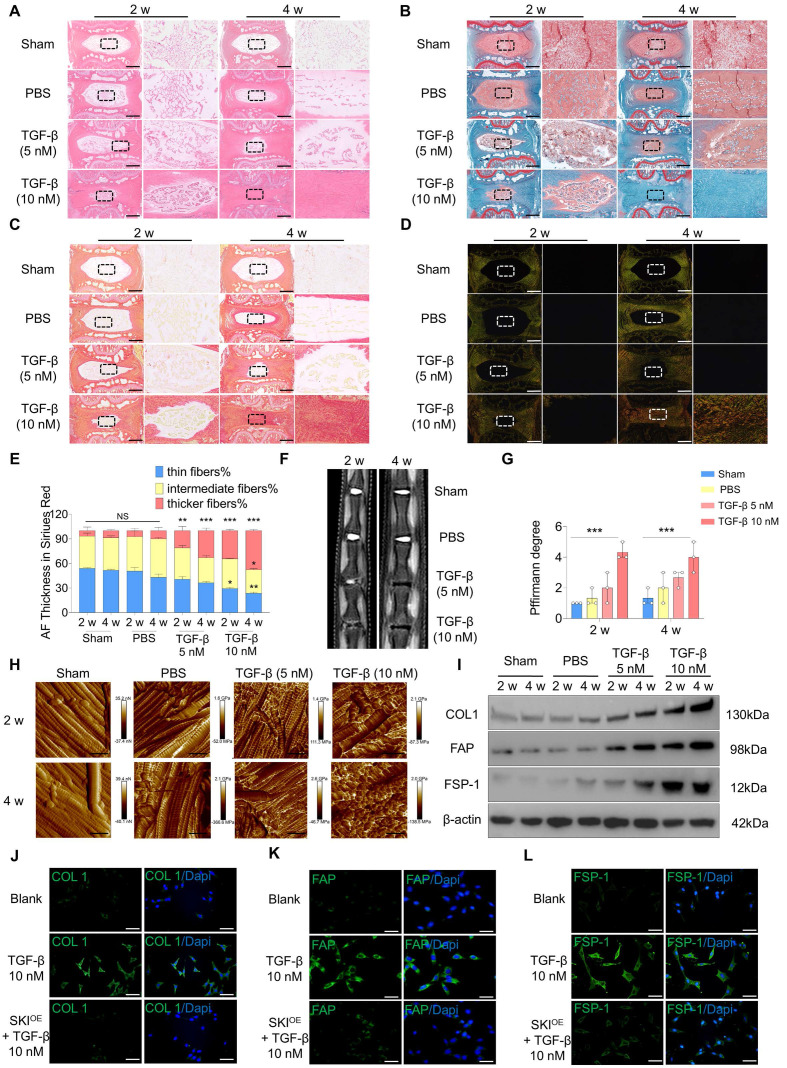
** Construction of *in vitro* and *in vivo* models of NP fibrosis. (A-B)** HE and SO-FO staining of rat caudal IVD sections 2 and 4 w Sham group or post-injection of 5 µl of PBS, 5 nM TGF-β or 10 nM TGF-β via a microsyringe. Scale bar = 200 µm, n = 3. **(C-E)** White light and polarized light Sirius red staining and statistical analysis of IVD sections 2 and 4 w Sham group or post-injection of 5 µl of PBS or 5 nM or 10 nM TGF-β via a microsyringe. Scale bar = 200 µm, n = 3, statistical differences were determined by two-way ANOVA, ***P < 0.001, **P < 0.01, *P < 0.05, NS: no significance. **(F-G)** MRI of caudal IVDs and statistical analysis of Pfirrmann grades 2 and 4 w Sham group or post-injection of 5 µl of PBS, 5 nM TGF-β or 10 nM TGF-β via a microsyringe, n = 3, statistical differences were determined by two-way ANOVA, ***P < 0.001. **(H)** Young's modulus of rat caudal IVDs measured by AFM. Scale bar = 400 nm, n = 3. **(I)** Western blotting analysis of COL1, FAP and FSP-1 protein expression in the NP tissue, n = 3. **(J-L)** Immunofluorescence of COL1, FAP and FSP-1 (green) expression 7 d post-induction with 10 nM TGF-β. The nuclei are shown enlarged and stained with DAPI (blue). Scale bar = 50 µm, n = 3.

To validate the role of TGF-β signaling activation in NP fibrosis, we injected 5 µl of TGF-β (5 nM), TGF-β (10 nM) or PBS into rat caudal IVDs. Sham group served as the blank control. Histological evaluation revealed that IVDD occurred in the NP tissue of the TGF-β group but not in that of the PBS group and Sham group at 2 and 4 w post-injection. Injection of 10 nM TGF-β resulted in more severe fibrosis, as shown by hematoxylin and eosin (HE) and safranin O-fast green (SO-FO) staining **(Figure 2A-B; Figure S2A)**. Sirius red staining revealed that the constituent ECM in the NP tissue of the TGF-β group transformed from thin fibers to intermediate and thicker fibers, and this transformation was more distinct post-injection of 10 nM TGF-β **(Figure 2C-E)**. Magnetic resonance imaging (MRI) revealed that the degree of degeneration increased significantly post-injection of TGF-β **(Figure 2F-G)**. X-ray images revealed a significant decrease in the intervertebral disc space in the TGF-β group, with a greater change in DHI% compared to both the PBS and Sham groups **(Figure S2B-C)**. Atomic force microscopy (AFM) was employed to evaluate the structural properties and Young's modulus of the NP tissue. The NP tissue in the PBS group presented regularly distributed collagen fibers and a low Young's modulus, but post-injection of TGF-β, the collagen fibers became disorganized and the Young's modulus increased, especially in the 10 nM TGF-β group **(Figure 2H; Figure S2D)**. The protein expression levels of collagen type I (COL1), FAP and fibroblast specific protein-1 (FSP-1), encoded by *COL1A1*, *FAP*, and *S100A4*, were significantly increased in the NP tissue post-injection of TGF-β **(Figure 2I; Figure S2E-G).** The RNA expression level of *TGFB1* was increased in the NP tissue post-injection of TGF-β, whereas the *SKI* expression level decreased **(Figure S2H-I)**, which was similar to the alterations in the fibrotic NP tissue revealed by scRNA-seq analysis.

Next, we investigated whether *SKI*-mediated inhibition of TGF-β signaling activation could alleviate fibrosis in NPCs *in vitro*. The expression level of *SKI* was downregulated in cultured NPCs after 7 d of induction with 10 nM TGF-β, similar to the *in vivo* results **(Figure S2J)**. A *SKI*-overexpressing plasmid (SKI^OE^) was then constructed and added to the TGF-β-induced NPCs **(Figure S2K)**. The COL1, FAP and FSP-1 protein levels in cultured NPCs were significantly increased post-TGF-β induction but decreased after SKI^OE^ treatment **(Figure 2J-L; Figure S2L-R)**. The role of TGF-β signaling activation was further verified in a puncture-induced disc degeneration (PIDD)-induced NP fibrosis model. We found that the expression of *TGFB1* was also upregulated and that *SKI* was also downregulated in the PIDD model **(Figure S3A-F)**. These results indicate that TGF-β signaling activation and *SKI* downregulation contribute to NP fibrosis and that SKI overexpression has a therapeutic effect on NP fibrosis.

### Loading *SKI* mRNA into functionalized Exos for targeted delivery to fibrotic NPCs

**Figure 3:**
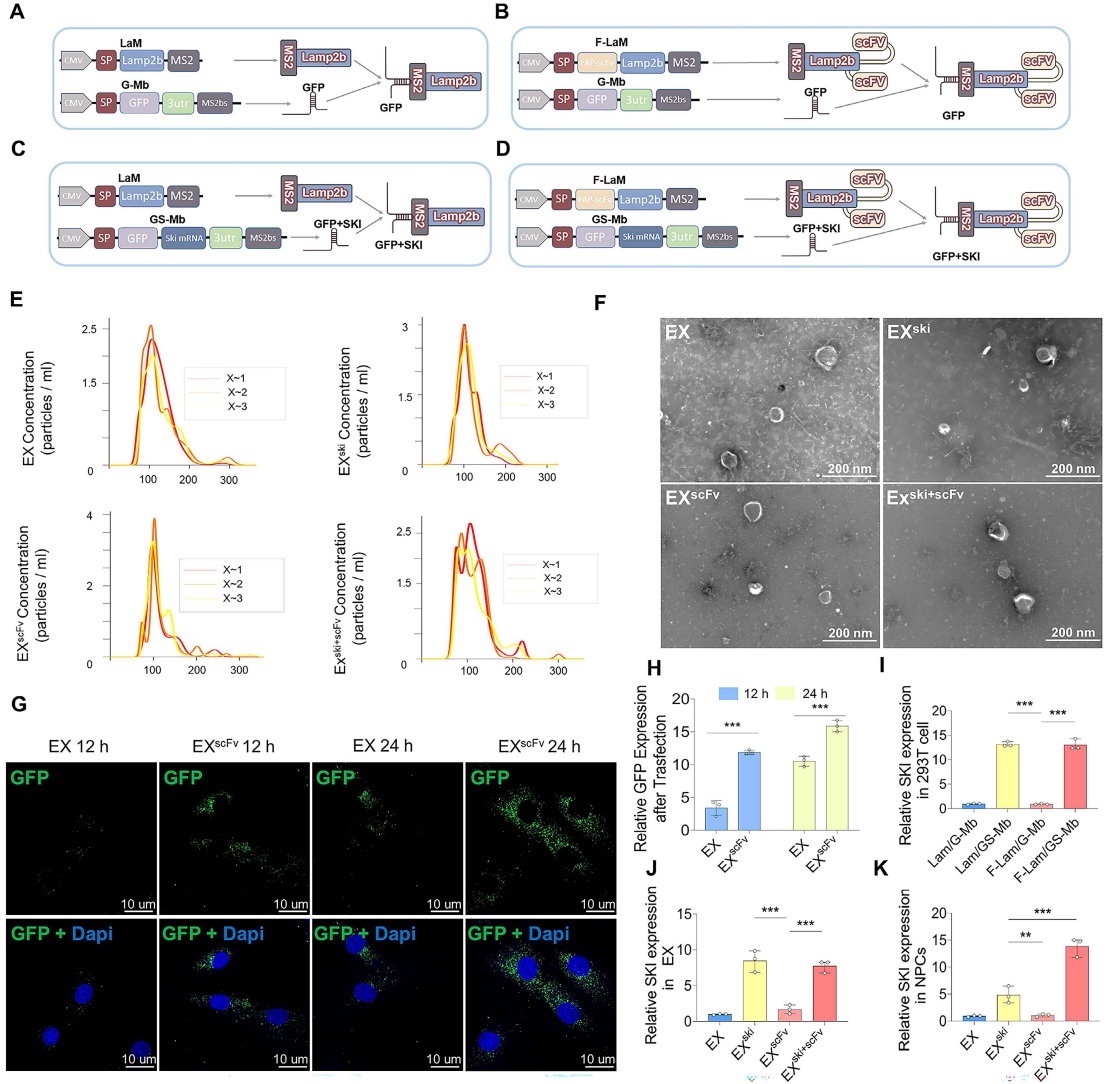
**Construction and synthesis of functionalized Exos. (A-D)** Scheme of EX, EX^scFv^, EX^ski^, and EX^ski+scFv^ construction according to the four plasmid combinations. **(E)** NTA analysis of Exos, n = 3. **(F)** Electron microscopy analysis of Exos, n = 3. **(G-H)** Immunofluorescence analysis of EX and EX^scFv^ (green) transfection efficacy at 12 and 24 h to analyze the targeting ability of Exos and perform statistical analysis. Nuclei were counterstained with DAPI (blue), statistical differences were determined by two-way ANOVA, n = 3, ***P < 0.001. **(I)** PCR assessment of *SKI* expression in HEK-293T cells after plasmid transfer, n = 3, statistical differences were determined by one-way ANOVA, ***P < 0.001. **(J)** PCR assessment of *SKI* expression in Exos, n = 3, statistical differences were determined by one-way ANOVA, ***P < 0.001. **(K)** PCR was used to assess *SKI* expression in NPCs. n = 3, statistical differences were determined by one-way ANOVA, ***P < 0.001, **P < 0.01.

To achieve stable *SKI* expression and fibrotic NPC-targeting properties, we constructed Exos functionalized with the scFv-LAMP2b-MS2-mRNA complex structure. Given that the membrane protein FAP can be the scFv target according to previous studies 32, 33 and that FAP is highly expressed in fibrotic NPCs, as shown in the sc-RNAseq results, FAP was chosen as the scFv target in our study. Four plasmids were constructed: one with the RNA bacteriophage coat protein MS2 inserted at the N-terminus of the vesicle-enriched protein LAMP2b (LaM); one with FAP-scFv fused to the N-terminus of LAMP2b-MS2 (F-LaM); one control plasmid with a green fluorescent protein (GFP) label and integrated homologous stem ring sequence MS2 binding sites (MS2bs) fused at the 3' end (G-Mb) that can bind MS2 via its specific stem-loop structure 34; and one with a GFP-*SKI* mRNA and MS2bs fusion (GS-Mb) **(Figure S4A)**. We speculated that the specific binding between MS2 and MS2bs would enable the *SKI* mRNA to be recruited into the functionalized Exos modified with FAP-scFv **(Figure 3A‒D)**. The plasmid pairs LaM/G-Mb, F-LaM/G-Mb, LaM/GS-Mb, and F-LaM/GS-Mb were cotransfected into HEK-293T cells, and the corresponding EX, EX^scFv^, EX^ski^, and EX^ski+scFv^ were harvested. Nanoparticle tracking analysis (NTA) revealed that most of the Exos were 50-200 nm in diameter **(Figure 3E)**, and transmission electron microscopy (TEM) revealed that all four types of Exos had a typical saucer-like double-layer membrane structure **(Figure 3F)**. The western blotting results revealed that all four types of Exos highly expressed the positive exosomal markers CD81 and TSG101 and weakly expressed the negative exosomal marker Calnexin **(Figure S4B-C)**. The immunofluorescence results revealed that more EX^scFv^ than EX were taken up by fibrotic NPCs induced by TGF-β **(Figure 3G-H)**. PCR analysis revealed that post-transfection with LaM/GS-Mb and F-LaM/GS-Mb, HEK-293T cells expressed higher levels of *SKI* than did the LaM/G-Mb- or F-Lam/G-Mb-transfected cells **(Figure 3I)**. The corresponding Exos, EX^ski^ and EX^ski+scFv^, also resulted in greater expression of *SKI* than did EX or EX^scFv^ **(Figure 3J)**, whereas the EX^ski+scFv^-treated NPCs presented higher *SKI* expression levels than did the NPCs treated with EX, EX^scFv^, or EX^ski^ **(Figure 3K)**. The results showed that functionalized Exos were successfully constructed to deliver *SKI* mRNA to fibrotic NPCs.

### Construction and characterization of a pH-Responsive exosome/hydrogel system

**Figure 4:**
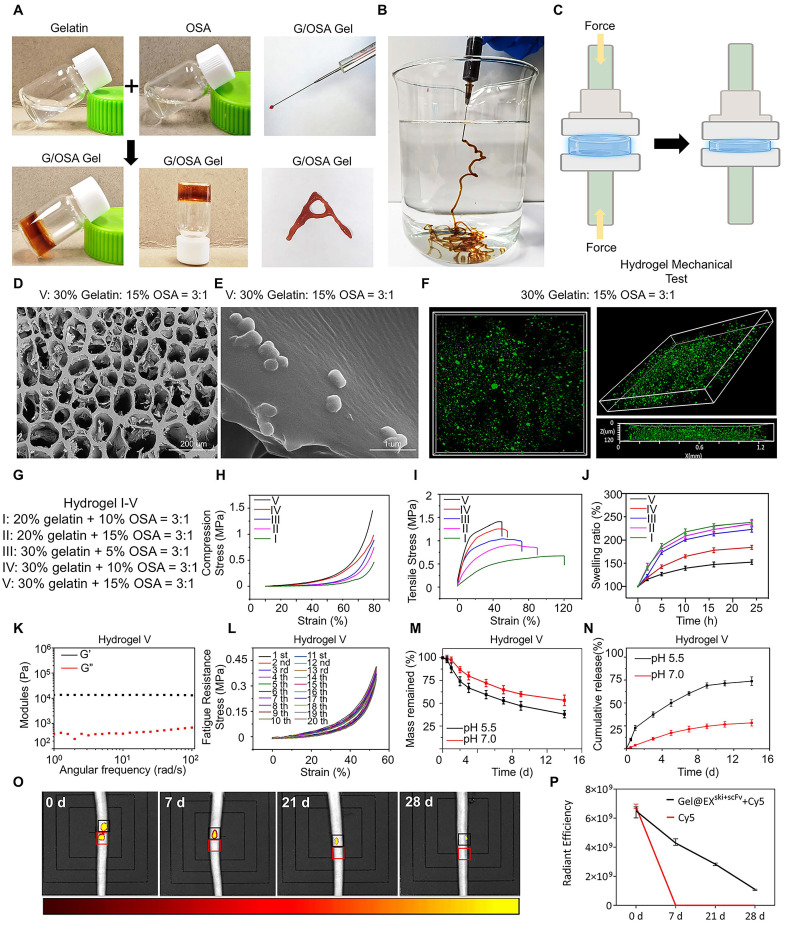
** Construction and biological and mechanical characterization of the exosome/hydrogel system. (A)** Scheme of how OSA and gelatin were used to synthesize the hydrogels. **(B)** Injectability of the hydrogel. **(C)** Schematic diagram of the hydrogel compression test. **(D)** Scanning electron microscopy images of the hydrogels. n = 3. **(E)** Environmental scanning electron microscope images of the hydrogels, n = 3, **(F)** Three-dimensional confocal image of hydrogel-encapsulated Exos (green), n = 3. **(G)** I-V hydrogel synthesis ratios. **(H)** Compression tests of five hydrogels with different ratios of OSA to gelatin, n = 3. **(I)** Tensile strength test of five hydrogels with different ratios of OSA to gelatin, n = 3. **(J)** Swelling rates of five hydrogels with different ratios of OSA to gelatin in PBS, n = 3. **(K)** Rheological properties of hydrogel V, n = 3 **(L)** Fatigue resistance of hydrogel V. **(M)** Degradation curves of hydrogel V at pH 7.0 and 5.5, n = 3. **(N)** Release curves of Exos from hydrogel V at pH 7.0 and 5.5, n = 3. **(O-P)** Hydrogel degradation *in vivo* and statistical analysis, n = 3.

A pH-responsive gelatin-OSA hydrogel was constructed. The aldehyde groups of OSA were obtained by oxidizing sodium alginate with sodium periodate, and ^1^H nuclear magnetic resonance (NMR) spectroscopy revealed new peaks in the OSA samples from 5.0-5.5 ppm and at 8.17 ppm compared with the spectra of the SA samples, suggesting the formation of aldehyde groups **(Figure S5A-B)**. Fourier transform infrared (FTIR) spectral analysis revealed the characteristic absorption peak of the aldehyde group at 1725 cm^-1^, confirming the successful introduction of the aldehyde group into the OSA chain **(Figure S5C)**. Mixing the flow dynamic OSA solution with the gelatin solution at room temperature resulted in rapid hydrogel formation within 10 s, which displayed satisfactory shape adaptability and injectability **(Figure 4A-B)**. Five hydrogels were prepared with different proportions of gelatin and OSA: I: 20% gelatin:10% OSA = 3:1; II: 20% gelatin:15% OSA = 3:1; III: 30% gelatin:5% OSA = 3:1; IV: 30% gelatin:10% OSA = 3:1; and V: 30% gelatin:15% OSA = 3:1. All the hydrogels had clear porous structures with uniform pore sizes, as observed by scanning electron microscopy, and as the solubility of the two-component solution increased, the pore size and porosity ratio of the hydrogels decreased **(Figure 4D; Figure S5D-F)**. Environmental scanning electron microscopy (ESEM) revealed that the microporous surface of the EX^ski+scFv^-loaded hydrogels (Gel@EX^ski+scFv^) was embedded with granular nanoparticles **(Figure 4E)**. Three-dimensional confocal imaging revealed that the GFP-labeled EX^ski+scFv^ in the hydrogel was distributed homogeneously **(Figure 4F)**. These changes in the internal network structure indicated that increasing the concentrations of OSA and gelatin led to higher aldehyde and amino group contents, which resulted in the formation of more dynamic bonds and effectively increased the degree of crosslinking of the hydrogel.

The compressive moduli of hydrogels I-V were 0.46, 0.74, 0.89, 0.98, and 1.45 MPa, and the compression modulus also increased from I-V. These increases may be attributed to the increasing contents of aldehyde groups and amino groups and the formation of more Schiff bases, which further increased the degree of crosslinking **(Figure 4H; Figure S5G)**. Moreover, the tensile modulus increased with increasing concentrations of OSA and gelatin, with the tensile modulus of hydrogel V reaching 1.45 MPa, indicating that increasing the Schiff base content further strengthened the degree of crosslinking of the hydrogel **(Figure 4I; Figure S5H)**. After immersion in PBS for 24 h, hydrogel V had the lowest swelling rate among all the hydrogels (152.5%), indicating that this hydrogel could better resist swelling and was suitable for IVD implantation** (Figure 4J)**. The rheological properties of hydrogel V were measured, and the storage modulus (G') of the hydrogel was greater than the loss modulus (G"), indicating that the sample was in an elastic solid-state and that its internal network structure remained intact under high angular frequency oscillations **(Figure 4K)**. The fatigue resistance results revealed that hydrogel V recovered rapidly and exhibited good fatigue resistance after 20 cycles, with a compressive strain greater than 50% **(Figure 4L)**. Hydrogel V was placed in PBS solutions with pH values of 5.5 and 7.0, and it degraded faster in the pH 5.5 solution than in the pH 7.0 solution **(Figure 4M)**. EX^ski+scFv^ was loaded in hydrogel V and named Gel@EX^ski+scFv^. The hydrogel was coincubated in solutions with different pH values, after which the solutions were collected and the release of fluorescent Exos was examined **(Figure S5I-J)**. More Exos were released from the hydrogels in PBS at pH 5.5 than in PBS at pH 7.0 at all the examined time points, particularly at 14 d, indicating pH-responsive release behavior **(Figure 4N)**. Then, rat caudal IVDs were treated with Cy5 alone or with Cy5 in Gel@EX^ski+scFv^. Free Cy5 was completely degraded *in vivo* by 7 d post-administration, whereas the Cy5 incorporated in Gel@EX^ski+scFv^ degraded by approximately 28 d post-administration **(Figure 4O-P)**.

**Figure 5:**
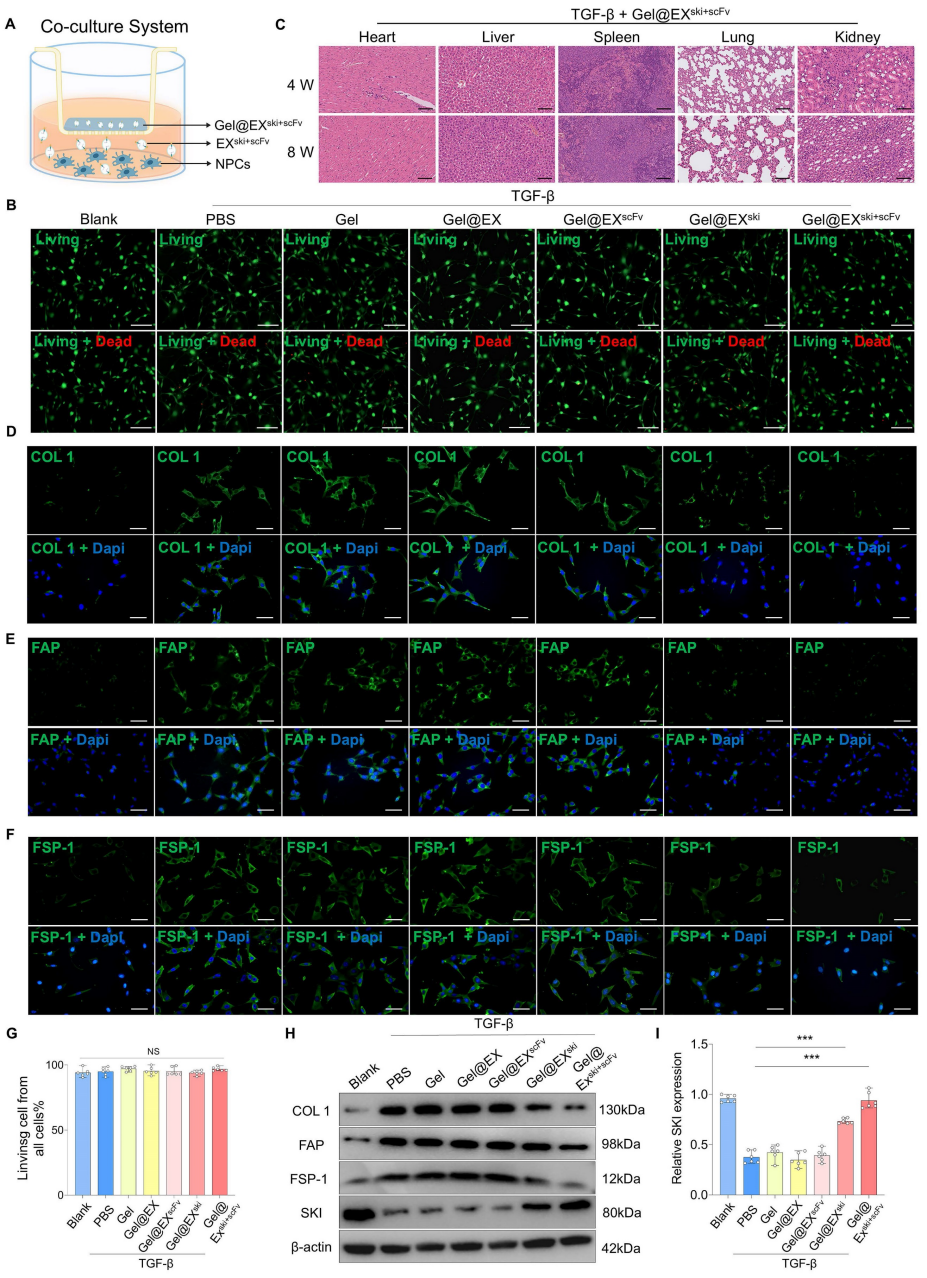
** Verification of the biocompatibility and therapeutic effect of Gel@EX^ski+scFv^. (A)** Schematic of the coculture system involving Gel@EX^ski+scFv^ and NPCs. **(B)** Live (green)/dead (red) cell staining assays. NPCs were cocultured with different exosome/hydrogel systems post-TGF-β induction. Scale bar = 100 µm, n = 6.** (C)** HE staining of the organs of the rats at 4 and 8 w post-injection of Gel@EX^ski+scFv^ and TGF-β induction. Scale bar = 50 µm, n = 6. **(D-F)** Immunofluorescence analysis of the expression of COL1 (green), FAP (green) and FSP-1 (green) in different exosome/hydrogel systems cocultured with NPCs after TGF-β-induced fibrosis. Nuclei were counterstained with DAPI (blue). Scale bar = 50 µm, n = 6. **(G)** Statistical analysis of live/dead cell staining assays, n = 6, statistical differences were determined by one-way ANOVA, NS: no significance. **(H-I)** Western blotting analysis of COL1, FAP, FSP-1 and SKI protein expression in NPCs cocultured with different exosome/hydrogel systems after TGF-β induction and SKI statistical analysis. n = 6, statistical differences were determined by one-way ANOVA, ***P < 0.001.

To verify the biocompatibility of the exosome/hydrogel system *in vitro*, Gel alone, Gel@EX, Gel@EX^ski^, and Gel@EX^ski+scFv^ were separately added to cultured NPCs treated with TGF-β in a coculture system **(Figure 5A)**. Live and dead staining and CCK8 assays revealed that Gel alone, Gel@EX, Gel@EX^ski^, and Gel@EX^ski+scFv^ were biologically safe for the treatment of NPCs *in vitro* **(Figure 5B; Figure 5G; Figure S6A)**. To verify the biocompatibility of the exosome/hydrogel system* in vivo*, Gel@EX, Gel@EX^ski^, and Gel@EX^ski+scFv^ were injected into rat caudal IVDs. The body weights of the rats did not significantly differ among the groups throughout the experiments **(Figure S6B)**. Blood samples were obtained, and the results revealed that after the injection of 10 µl of Gel@EX^ski+scFv^, the hematological and blood chemistry parameters did not significantly differ from those of the groups injected with PBS **(Table S1-2)**. At 8 w post-injection, the organs, including the heart, liver, spleen, lung and kidney, of the rats were isolated and weighed, and the results revealed that there were no significant differences in the weights of different organs among the groups **(Figure S6C-G)**. HE staining was performed on the heart, liver, spleen, lung and kidney of the rats at 4 and 8 w post-treatment, and the results revealed no apparent toxicity of the exosome/hydrogel system **(Figure 5C; Figure S6H)**.

To further verify the biosafety of the exosome/hydrogel system, we implanted the exosome/hydrogel system subcutaneously in rats. Blood samples were collected from the rats at 4 w for hematological and blood chemistry parameters. The results showed no significant differences compared to the Sham group **(Table S3-4)**. The body and organs of the rats were weighed and the organs were performed HE staining. The results indicated that there were no significant differences in the body weight and organ-to-body weight ratios among the groups at 4 w **(Figure S7A-F)**. The HE staining suggested no toxicity in the organs after subcutaneous implantation of the exosome hydrogel system **(Figure S7G)**.

### Verification of the antifibrotic effect of Gel@EX^ski+scFv^ *in vitro* and *in vivo*

Gel alone, Gel@EX, Gel@EX^ski^, and Gel@EX^ski+scFv^ were separately added to TGF-β-treated NPCs in a coculture system. The immunofluorescence results suggested that the COL1, FAP and FSP-1 expression levels were significantly increased in the TGF-β group, and both the Gel@EX^ski^ and Gel@EX^ski+scFv^ groups presented decreased COL1, FAP and FSP-1 levels, but Gel@EX^ski+scFv^ presented better therapeutic effects than did Gel@EX^ski^ **(Figure 5D-F, Figure S8A-C)**. The western blotting results also revealed that the Gel@EX^ski+scFv^-treated NPCs presented the lowest COL1, FAP and FSP-1 expression and the highest SKI protein expression **(Figure 5H-I; Figure S8D-F)**.

**Figure 6:**
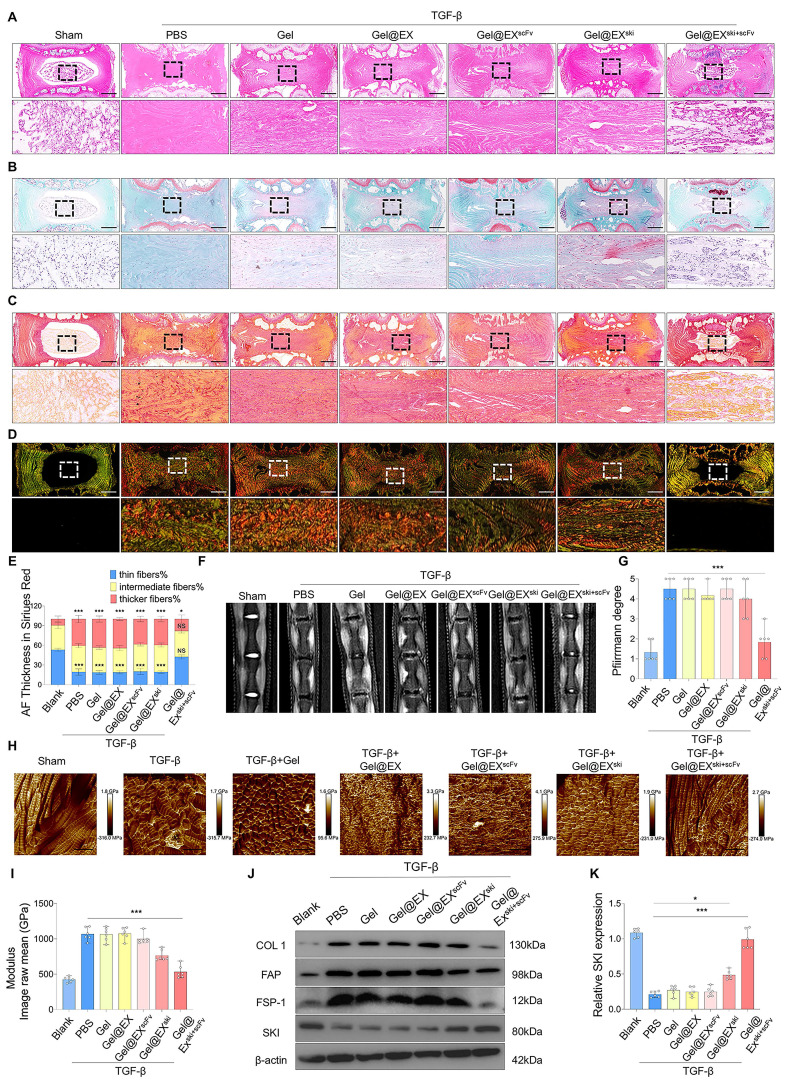
**Verification of the *in vivo* therapeutic effect of Gel@EX^ski+scFv^. (A-B)** HE staining and SO-FO staining post-injection of 10 µl of different exosome/hydrogel mixtures after TGF-β-induced NP fibrosis. Scale bar = 200 µm, n = 6. **(C-E)** Sirius red staining and white light and polarized light analysis were used to evaluate the changes in the thickness of the NP fibers post-injection of 10 µl of different exosome/hydrogel systems after TGF-β-induced NP fibrosis. Scale bar = 200 µm, n = 6, statistical differences were determined by two-way ANOVA, ***P < 0.001, *P < 0.05, NS: no significance. **(F-G)** MRI of the changes in the Pfirrmann scores of the NP tissues post-injection of 10 µl of different exosome/hydrogel systems after TGF-β-induced NP fibrosis and statistical analysis. n = 6, statistical differences were determined by one-way ANOVA, ***P < 0.001. **(H-I)** Changes in the Young's modulus of the NP tissues post-injection of 10 µl of different exosome/hydrogel systems after TGF-β-induced NP fibrosis and statistical analysis. Scale bar = 400 nm, n = 6, statistical differences were determined by one-way ANOVA, ***P < 0.001. **(J-K)** Western blotting analysis of COL1, FAP, FSP-1 and SKI protein expression post-treatment with different exosome/hydrogel systems after TGF-β-induced NP fibrosis and SKI statistical analysis. n = 6, statistical differences were determined by one-way ANOVA, ***P < 0.001, *P < 0.05.

The antifibrotic effects of Gel@EX^ski+scFv^ were further explored *in vivo*. At 4 w post-injection, HE and SO-FO staining revealed no significant histopathological alterations in the Gel alone or Gel@EX groups compared with the TGF-β group, and the Gel@EX^ski+scFv^ group presented distinctly lower histological scores than those of the TGF-β group** (Figure 6A-B; Figure S9A)**. Sirius red staining revealed fewer thick fibers and more thin fibers in the Gel@EX^ski+scFv^ group than in the other groups **(Figure 6C-E)**. MRI and X ray revealed distinctly lower Pfirrmann scores and higher DHI% changes in the Gel@EX^ski+scFv^ group than in the TGF-β group** (Figure 6F-G; Figure S9B-C)**. The AFM results revealed that the Gel@EX^ski+scFv^ group presented more regularly distributed collagen fibers and a lower Young's modulus **(Figure 6H-I)**. The western blotting results revealed that the NP tissue of the Gel@EX^ski+scFv^ group presented the highest SKI expression level and distinctly reduced COL1, FAP and FSP-1 expression levels among all the groups **(Figure 6J-K; Figure S9D-F)**. These results suggest that Gel@EX^ski+scFv^ effectively elevated the SKI expression level in the NP tissue and alleviated NP fibrosis.

### SKI protein alleviated NP fibrosis by interacting with FOXO3 to regulate TGF-β/AKT/FOXO3 signaling

**Figure 7:**
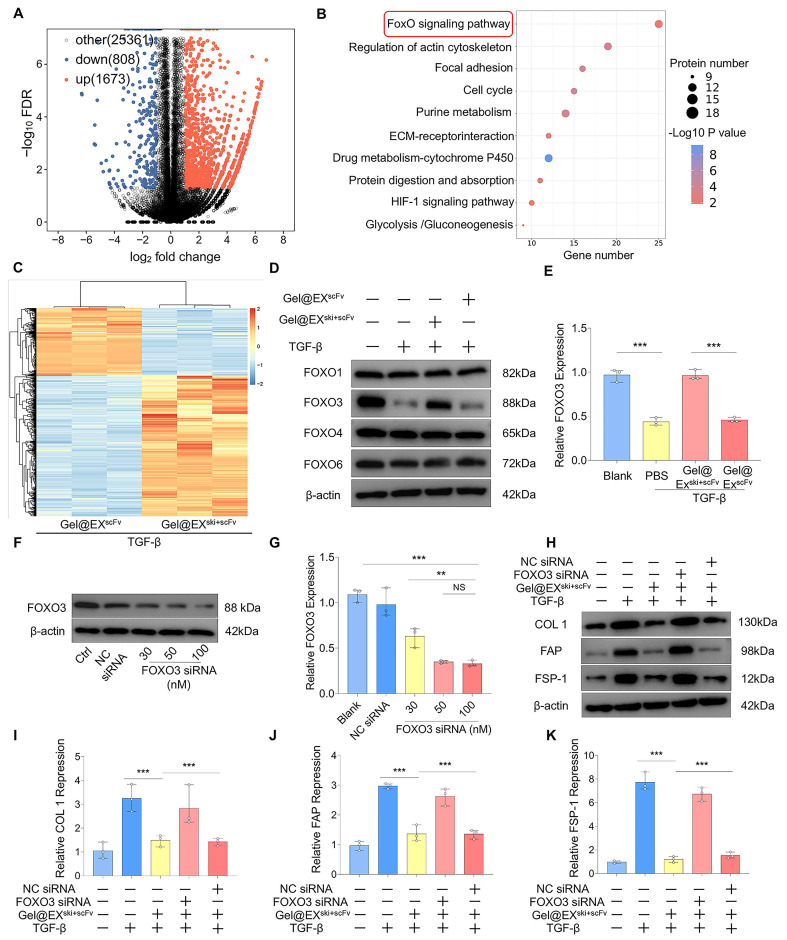
**Exploration of the mechanism by which *SKI* overexpression inhibits fibrosis in NPCs. (A-C)** RNA-sequencing analysis of NPCs treated with Gel@EX^scFv^ or Gel@EX^ski+scFv^ post-TGF-β induction. n = 3. **(D-E)** Western blotting analysis of the changes in FOXO1, FOXO3, FOXO4, and FOXO6 expression and statistical analysis of FOXO3 expression. n = 3, statistical differences were determined by one-way ANOVA, ***P < 0.001. **(F-G)** Western blotting analysis of FOXO3 expression post-siRNA transfection and statistical analysis. n = 3, statistical differences were determined by one-way ANOVA, ***P < 0.001, **P < 0.01, NS: not significant. **(H-K)** Western blotting analysis of FOXO3 siRNA-mediated inhibition of the antifibrotic effect of Gel@EX^ski+scFv^ and statistical analysis. n = 3, statistical differences were determined by one-way ANOVA, ***P < 0.001.

To explore the mechanism of SKI in NP fibrosis, we performed RNA sequencing on TGF-β-induced NPCs treated with Gel@EX or Gel@EX^ski+scFv^ post-TGF-β induction. A total of 1863 genes were upregulated and 808 were downregulated in the Gel@EX^ski+scFv^ group compared with the Gel@EX group **(Figure 7A)**. KEGG analysis revealed that the differentially expressed genes (DEGs) were enriched in the FOXO pathway **(Figure 7B)**. Western blotting assays revealed that the expression level of FOXO3 decreased post-TGF-β induction but increased after Gel@EX^ski+scFv^ treatment, whereas the expression levels of other FOXO proteins were not distinctly altered **(Figure 7D-E; Figure S10A‒C)**. The expression of FOXO3 was subsequently knocked down by siRNA **(Figure 7F-G)**, abolishing the beneficial effects of Gel@EX^ski+scFv^ in TGF-β-induced NPCs **(Figure 7H‒K)** and indicating that the antifibrotic effects of *SKI* overexpression were related to FOXO3.

**Figure 8:**
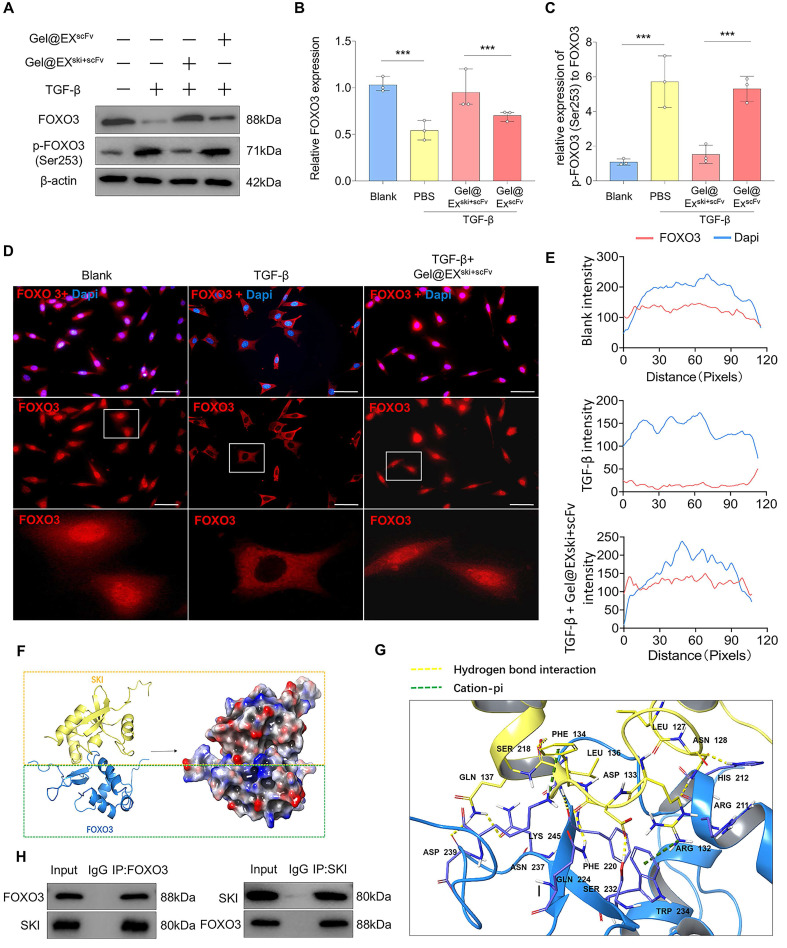
**Exploration of the interaction between the SKI protein and the FOXO3 protein. (A-C)** Western blotting analysis of FOXO3 and p-FOXO3 expression and statistical analysis. n = 3, statistical differences were determined by one-way ANOVA, ***P < 0.001. **(D-E)** Immunofluorescence verification that FOXO3 (red) was exported from the nucleus upon TGF-β induction and entered the nucleus via Gel@EX^ski+scFv^ and statistical analysis of FOXO3 nuclear fluorescence; the nuclei were stained with DAPI (blue). Scale bar = 50 µm. **(F-G)** Molecular docking revealing the interaction between the FOXO3 protein and the SKI protein. **(H)** Co-IP analysis revealed that the FOXO3 protein could bind the SKI protein.

TGF-β can regulate the function of FOXO3 via AKT-mediated FOXO3 phosphorylation, which then promotes FOXO3 nuclear export and degradation 35, 36. Therefore, the levels of phosphorylated AKT (p-AKT) and phosphorylated FOXO3 (p-FOXO3) were determined. The western blotting results revealed that FOXO3 phosphorylation increased post-TGF-β induction but decreased after Gel@EX^ski+scFv^ treatment **(Figure 8A‒C)**. Moreover, AKT phosphorylation increased post-TGF-β induction but was not altered after Gel@EX^ski+scFv^ treatment, indicating that Gel@EX^ski+scFv^ regulated TGF-β signaling by regulating the phosphorylation of FOXO3 **(Figure S11A‒D)**. The immunofluorescence results revealed that the nuclear FOXO3 level decreased post-TGF-β induction but increased after Gel@EX^ski+scFv^ treatment **(Figure 8D-E)**. Furthermore, molecular docking revealed that SKI directly interacts with FOXO3 **(Figure 8F-G)**, and this binding interaction was verified by coimmunoprecipitation experiments **(Figure 8H)**. These results showed that Gel@EX^ski+scFv^ repressed TGF-β signaling by the interaction of SKI with FOXO3 and the inhibition of FOXO3 phosphorylation.

## Discussion

Fibrosis is the pathological result of abnormal tissue repair and remodeling, especially in chronic degenerative and inflammatory diseases 37. The ECM in healthy NP tissue is composed of proteoglycans and collagen type II, which results in abundant water and viscoelasticity in IVDs to absorb mechanical loads. NP fibrosis is one of the mechanisms of compensatory repair in the early stage of IVDD 38. However, as IVDD progresses, the compensatory repair mechanism is overactivated, resulting in ECM deposition and collagen remodeling, which induce NP tissue destruction and dysfunction. Previous studies have shown that inhibiting NP fibrosis can alleviate IVDD 39-41. Fibrotic tissues often show strong heterogeneity in which fibrotic cells and normal cells coexist; thus, precise and targeted treatments for fibrotic diseases are being investigated 42-44. At present, reports on NP fibrosis therapy have not provided accurate targeted treatment, which is partly due to the unclear mechanism of NP fibrosis. Therefore, scRNA-seq was used to analyze fibrotic NPC subsets in NP fibrosis and explore the role of TGF-β signaling. Thus, we designed the Gel@EX^ski+scFv^ system that targets FAP in fibrotic NPCs for the precise delivery of *SKI* mRNA. The results showed that Gel@EX^ski+scFv^ can effectively alleviate NP fibrosis and IVDD. Mechanistically, the SKI protein inhibits NP fibrosis by regulating TGF-β/AKT/FOXO3 signaling by binding to the FOXO3 protein. These findings provide a novel strategy for the accurate and effective treatment of NP fibrosis.

ScRNA-seq has been widely used to define fibrotic cell subsets in fibrotic tissues and to analyze new therapeutic targets 45. Recent scRNA-seq studies have revealed fibrotic NPC subsets in IVDD 12, 46; therefore, we analyzed mild and severe degenerative NP via scRNA-seq. The results revealed the proportions of the fibrotic NPC subsets expressing the fibrotic markers *FAP*, *S100A4*, *COL1A1*, and *TGFB1*. In addition, the expression levels of *FAP*, *S100A4*, *COL1A1*, and *TGFB1* were increased in the fibrotic NPC subsets of severe degenerative NP tissue. Further analysis revealed that the DEGs were enriched in the TGF-β signaling pathway. TGF-β signaling is an important regulatory pathway in organ development, cell proliferation and differentiation, and tissue damage and repair. However, overactivation of TGF-β signaling can lead to fibrosis of the lung, liver, myocardium, skin, kidney and other tissues 47. High levels of TGF-β have been reported to induce fibrosis-like changes in NPCs *in vitro* 48. In our study, radiographical, histological, and cytobiological results revealed that *in vivo* TGF-β injection and *in vitro* TGF-β treatment directly induced NP fibrosis. Additionally, the activation of TGF-β signaling was further confirmed in a PIDD-induced fibrosis model. These results indicate that activation of the TGF-β pathway is related to the occurrence of NP fibrosis.

*SKI* is an important repressor of TGF-β signaling that can inhibit both classical and nonclassical TGF-β signaling. *SKI* has been shown to inhibit fibrosis in different tissues 19-22; however, the role of *SKI* in NP fibrosis has not been reported. ScRNA-seq analysis revealed that the expression level of *SKI* in fibrotic NPC subsets of severe degenerative NP tissue was lower than that in those of mild degenerative NP tissue. A decreased *SKI* expression level was also confirmed in PIDD-induced fibrosis *in vivo* and in TGF-β-induced NP fibrosis *in vitro*, suggesting that *SKI* may have the potential to treat NP fibrosis. In addition, we successfully inhibited TGF-β-induced NP fibrosis *in vitro* by using a plasmid overexpressing *SKI*. However, *SKI* overexpression induced by plasmid transfection was not sustained and could not be achieved *in vivo*. In previous studies, most *in vivo SKI*-overexpression gene therapies have relied on adenoviruses, adeno-associated viruses, etc., and the potential inflammatory effects and toxic reactions after virus injection may affect the therapeutic outcomes of these treatments. Moreover, these gene therapies may lead to viral integration into the genome 23, 24.

Exo-based mRNA therapies offer the possibility of precisely delivering *SKI* mRNA. Since the advent of mRNA vaccines for the treatment of COVID-19, the methods by which mRNAs are carried by Exos have gradually been used for the treatment of various diseases 49, 50. mRNA can directly use the intracellular RNA translation pathway for protein synthesis, which has the advantage of transient expression. However, all mRNA-based drugs must enter the cytoplasm for translation, which is challenging because mRNA molecules are large and negatively charged, making it difficult for them to passively cross the negatively charged cell membrane. In addition, single-stranded mRNAs are extremely fragile, and various enzymes in blood and tissues can rapidly degrade mRNAs and induce innate immune responses 51. Exos are promising delivery vehicles for mRNAs because of their inherent biocompatibility, ability to cross physiological barriers, and low immunogenicity. Moreover, the specific components of Exos can stabilize mRNAs, helping to prevent mRNA degradation by the extracellular barrier. We used an active preloading approach to load *SKI* mRNA into Exos with two plasmids for transfection. In one plasmid, LaM, the LAMP2b sequence was fused to the MS2 phage coat protein. In the second plasmid, GS-Mb, the MS2bs sequence bound to the MS2 protein was fused to the *SKI* mRNA sequence. This cotransfection method can promote not only the expression of mRNAs in Exo-producing cells but also the loading of mRNAs into Exos 52. Our results showed that this loading method successfully resulted in *SKI* mRNA overexpression in EX^ski^ secreted by HEK-293T cells transfected with both plasmids.

Based on these findings, we planned to deliver *SKI* precisely and efficiently to fibrotic NPC subsets via Exo functionalization for the treatment of NP fibrosis. ScRNA-seq revealed that fibrotic NPC subsets highly expressed FAP, a cell membrane protein expressed in fibrotic cells 53, 54 that has also been used for delivering drugs for fibrosis-targeted therapy 55, 56. FAP has also been identified as a fibrotic marker in several studies of NP fibrosis 3, 57. At present, the use of FAP-based scFvs targeting FAP-positive tumor-associated fibroblasts has been reported in the field of cancer therapy 32, 33. Moreover, surface modification of Exos with scFvs has been shown to confer upon Exos the ability to target specific cells or specific cell surface proteins 58, 59. The FAP-scFv sequence was fused to the vesicle-enriched protein LAMP2b and then loaded with MS2 on the phage coat protein to obtain the improved recombinant plasmid F-LaM. HEK-293T cells were cotransfected with F-LaM and GS-Mb to extract EX^ski+scFv^, which improved the targeting of fibrotic NPCs post-TGF-β induction and increased *SKI* mRNA expression in fibrotic NPCs after coculture.

To further promote the sustained release of EX^ski+scFv^ for the treatment of IVDD, we designed pH-responsive hydrogels loaded with EX^ski+scFv^ for sustained Exo release. After mixing the flow dynamic OSA with the gelatin solution at room temperature to form G/OSA, EX^ski+scFv^ was blended with G/OSA to obtain Gel@EX^ski+scFv^. The mechanical tests revealed that when 30% gelatin and 15% OSA were mixed at a ratio of 3:1, the compression modulus peaked at 1.45 MPa, which was due to the increased contents of aldehyde groups and amino groups, the formation of Schiff bases and the degree of crosslinking, which further strengthened the hydrogel structure 60. The results of the compression cycle test revealed that the hydrogel shape recovered rapidly, and the elasticity and fatigue resistance were good when the compressive strain was greater than 50% for 20 cycles. This occurred because the dynamic bonds in the hydrogel break when pressure is applied to disperse the external force, whereas after the pressure is removed, the dynamic bonds are regenerated so that the shape of the hydrogel is restored, and the internal structure changes very little 61. The hydrogel also exhibited a better tensile modulus because the increase in the Schiff base content further strengthened the degree of crosslinking of the hydrogel and improved the tensile strength. Degradation and Exo release experiments in PBS at different pH values revealed that Gel@EX^ski+scFv^ exhibited better pH-responsive degradation characteristics and sustained Exo release. The degradation trend of Gel@EX^ski+scFv^ and the release trend of Exos were consistent with the pH-responsive characteristics of the Schiff base. In an acidic environment, the Schiff base is cleaved, reducing the degree of crosslinking of the hydrogel, causing Gel@EX^ski+scFv^ to degrade more rapidly and allowing Exos to be released from the hydrogel on demand.

We further conducted* in vitro* and *in vivo* studies using Gel@EX^ski+scFv^ to examine its therapeutic effect on NP fibrosis. Coculture experiments, live and dead assays and CCK8 assays revealed that Gel@EX^ski+scFv^ had no significant toxicity to NPCs. HE staining, organ weights and body weights indicated ideal biocompatibility. Gel@EX^ski+scFv^ treatment further reduced the expression levels of fibrotic marker proteins in NPCs, which were greater than those in the Gel, Gel@EX, Gel@EX^ski^, and Gel@EX^scFv^ groups. These findings indicate that Gel@EX^ski+scFv^ has good targeting ability and can effectively inhibit NP fibrosis *in vitro*. We further evaluated the therapeutic effect of Gel@EX^ski+scFv^ on TGF-β injection-induced NP fibrosis *in vivo*. The radiographical, histological, and cytobiological results revealed that Gel@EX^ski+scFv^ treatment resulted in the greatest alleviation of TGF-β-induced NP fibrosis. Actually, Gel, Gel@EX, Gel@EX^scFv^ showed no apparent amelioration in NP fibrosis and no apparent SKI overexpression, which may result from the absence of therapeutic SKI mRNA in the exosome/hydrogel systems. Gel@EX^ski^ resulted in slight alleviation in NP fibrosis and overexpression in SKI level, as shown by slightly ameliorated histological score, slightly elevated DHI%, slightly declined fibrotic proteins expression, possibly owing to its absence of targeted delivery ability for SKI mRNA. In comparison, Gel@EX^ski+scFv^ exhibited the most potent effect among all the groups in alleviating NP fibrosis and elevating SKI expression, as shown by ameliorated histological score and Pfirrmann score, elevated DHI%, reduced Young's modulus, and declined fibrotic proteins expression, possibly attributed to the synergistic effects of successful SKI expression by high-level SKI mRNA and effective targeted delivery mediated by FAP-scFv.

To define the potential mechanism of *SKI* mRNA delivery for the treatment of NP fibrosis, we performed RNA sequencing on NPCs after Gel@EX^ski+scFv^ intervention. The results revealed that the FOXO signaling pathway was enriched in the DEGs. The FOXO family, which includes the transcription factors FOXO1, FOXO3, FOXO4 and FOXO6, is important in regulating the cellular response to stimuli 62. FOXO1 and FOXO3 are considered inhibitors of fibrosis 63, 64. Western blotting analysis revealed that the expression of FOXO3 decreased after TGF-β intervention but increased significantly after Gel@EX^ski+scFv^ intervention. Further siRNA experiments confirmed that the effect of Gel@EX^ski+scFv^ treatment was inhibited after FOXO3 knockdown, suggesting that FOXO3 is an important downstream target of *SKI*-mediated inhibition of TGF-β-induced NP fibrosis. TGF-β signaling has been confirmed to mediate FOXO3 phosphorylation by promoting AKT phosphorylation, thereby inducing the translocation of FOXO3 from the nucleus to the cytoplasm for its subsequent degradation; notably, this pathway is an important regulatory pathway in nonclassical TGF-β signaling 35, 36. Our results revealed that the level of p-FOXO3 decreased and the nuclear content of FOXO3 increased after SKI was overexpressed, but the level of p-AKT did not change significantly, suggesting that SKI may play a role in inhibiting fibrosis by regulating the phosphorylation of FOXO3 directly. The molecular docking results revealed that the SKI protein can form hydrogen bonds with Asp233 and Lys245 of the FOXO3 protein, adjacent to the site of FOXO3 phosphorylation (Ser253) 36, which may be associated with the inhibition of FOXO3 phosphorylation by SKI. Coimmunoprecipitation confirmed that the SKI protein can interact with the FOXO3 protein. Overall, these results suggest that SKI can directly bind to FOXO3 and affect its phosphorylation to repress TGF-β-induced NP fibrosis.

Our study has several limitations. Firstly, detailed comparisons with alternative approaches, such as other delivery systems or regenerative therapies for nucleus pulposus fibrosis and IVDD is needed in the future work. Secondly, the sample size in our animal experiments is a little bit small to prove the good efficiency of the Gel@EX^ski+scFv^ system in preventing nucleus pulposus fibrosis. Thirdly, other nucleus pulposus fibrosis animal models should be utilized in the future experiments to verify whether Gel@EX^ski+scFv^ system can also have a good targeting capacity in other specific microenvironmental during IVDD. Finally, potential differences in materials behavior in larger animal models or humans are needed to further verify the efficiency and biosafety of the Gel@EX^ski+scFv^ system.

## Conclusion

In summary, our study revealed the roles of TGF-β signaling activation and the TGF-β signaling repressor *SKI* in fibrotic NPC subsets in NP fibrosis via scRNA-seq. Furthermore, using active preloading and a scFv-targeted delivery strategy, we successfully obtained functionalized Exos that can deliver *SKI* mRNA to target fibrotic NPC subsets upon scFv binding to FAP. Furthermore, the pH-responsive Gel@EX^ski+scFv^ delivery system can respond to the acidic environment in IVDs and achieve the sustained release of EX^ski+scFv^, which effectively reduces TGF-β-induced NP fibrosis *in vitro* and *in vivo*. Mechanistically, SKI may inhibit TGF-β/AKT/FOXO3 signaling by binding to FOXO3. These findings may provide a new strategy for promoting accurate and effective NP fibrosis treatment, thereby effectively alleviating IVDD in clinical practice.

## Methods

### scRNA-seq

The scRNA-seq data from the NP tissue were downloaded from the GEO database (GSE244889). Analysis of the expression matrix was completed using the R package Seurat (Version: 4.2.0), and the samples were preprocessed by excluding low-quality cells using the R package Harmony (Version: 0.1.1). The samples were then combined and projected onto a 2D space using the first 30 principal components using the UMAP function. The cells were then subjected to unsupervised clustering using the FindClusters function with a resolution of 0.5. The highly expressed genes relative to the other clusters were identified using the FindAllMarkers function with thresholds of log2FC > 0.25, P Adj < 0.05, and min.pct > 0.1. The cell types were then identified based on known marker genes. The NPC cluster was selected and reanalyzed using UMAP, unsupervised clustering, and differential expression analysis. GO term and KEGG pathway enrichment analyses were performed online using the DAVID website, and GSEA was performed using the R package clusterProfiler (Version: 4.4.4) with pathway gene sets obtained from the KEGG database.

The IVD NP subsets were as follows 46: fibro-NPCs, with the markers *ACAN*, *SOX9*, and *FBLN1*; IR-NPCs-1, with the markers *ACAN*, *SOX9*, and *CH3L236*; IR-NPCs-2, with the markers *ACAN*,* SOX9*, and* CHI3L1*; Adh-NPCs, with the markers *ACAN, SOX9*, and *MSMO136*; SR-NPCs, with the markers* ACAN, SOX9*, and *CP*; T cells, with the markers* CD3E* and *TRAC*; B cells, with the markers *CD79A, MS4A1* and *MZB1*; monocytes, with the markers *FCN1*,* CD300E*, and *CSF3R*; macrophages, with the markers *C1QA*,* TREM2*, and *MRC1*; endothelial cells, with the markers *PECAM1*,* VWF*, and *CDH5*; smooth muscle cells, with the markers *ACTA2*, *MYH11*, and *TAGLN*; and red blood cells, with the markers *HBB*,* HBA1,* and *CA1*.

### Animals and surgical procedures

All animal experiments were approved by the Ethics Committee of the Second Affiliated Hospital of the Army Medical University (SCXK2022-0011) and performed in accordance with the principles and procedures of the National Institutes of Health (NIH) Guidelines for Laboratory Animal Care and Use and the Army Medical University Guidelines for Animal Treatment. All the animals were raised under pathogen-free conditions at 26-28 °C with 50-65% humidity on a 12-h day/night cycle. All biological replicates in our *in vivo* experiments represent individual rats.

For the TGF-β injection-induced NP fibrosis model, SD rats aged 6-8 w (Experimental Animal Center, Army Medical University, Chongqing, China) were anesthetized using a mixture of ketamine and toluene thiazide (30 mg/kg ketamine and 12 mg/kg toluene thiazide). The caudal vertebrae were exposed, and 5 µl of TGF-β (5 nM or 10 nM) (Solarbio, China) was injected into IVDs using a 26-gauge microsyringe (Hamilton, Switzerland) with a pressure rating of 2000 psig (137.9 bar). The rats in the PBS group were injected with 5 µl of PBS. To verify the therapeutic effect and biological safety of the exosome/hydrogel system, 10 µl of the exosome/hydrogel system was injected, and the PBS group was injected with 10 µl of PBS after TGF-β-induced fibrosis. After injection, the skin was sutured and disinfected. Rat caudal vertebrae were obtained at designated time points. To further verify the biological safety of the hydrogel-exosome system, we implanted the hydrogel-exosome system (10 µl) subcutaneously in rats. After 4 w, the internal organs of the rats were harvested for weighing and subjected to HE staining to assess biological safety.

For the PIDD model, SD rats aged 6-8 w (Experimental Animal Center, Army Medical University, Chongqing, China) were anesthetized with a mixture of ketamine and toluene thiazide (30 mg/kg ketamine and 12 mg/kg toluene thiazide). The caudal vertebrae were exposed, and a 20-gauge needle was inserted into IVDs to a depth of approximately 5 mm. The needle was rotated 360 degrees for 30 s and then removed vertically, after which the skin was sutured and disinfected. Rat caudal vertebrae were obtained at designated time points.

### MRI

SD rats were anesthetized and subjected to MRI using a PHILIPS Ingenia 3.0T. The parameters were set as follows: TR time, 2000 ms; TE time, 80 ms; excitation time, 2; scanning time, 3 min, 20 s; fat compression technology, SPAIR; scanning matrix (FOV), 368 × 288; voxels, 0.3 × 0.3 × 2.5 mm; layer thickness, 2.5 mm; echo chains, 12; and spin echo sequence, TSE sequence. The mouse coil used was model no. MS120 (Medcoil Healthcare, China). The Pfirrmann grades of the rat IVDs were calculated according to the Pfirrmann grading criteria for the T1- and T2-weighted imaging parameters.

### X ray

After being anesthetized, the rats had their tails amputated for X-ray examination (Carestream Evolution, USA). The parameters of the X-ray machine are as follows: tube voltage of 55 kV, tube current of 6 mAs, and source-to-image distance (SID) of 140 cm. The length of the intervertebral disc and the adjacent superior and inferior vertebral bodies were measured to calculate the disc height index (DHI), and the percentage of DHI (DHI%) was calculated as post-DHI/pre-DHI.

### Histological staining

Fixed IVDs were embedded in paraffin and then subjected to histological sectioning (8-µm thickness). For histological assessment, the paraffin sections were deparaffinized in graded xylene, rehydrated in graded alcohol solutions, washed and stained with SO-FO and HE (Servicebio, Wuhan, China) per the manufacturer's protocols. The histological score was calculated based on the modified histologic grading system. For Sirius red staining, dehydrated paraffin sections were stained with Sirius red staining solution for 8 min. The samples were then placed in clean xylene for 5 min, sealed with neutral gum, and examined under a microscope for image collection and analysis.

### AFM analysis

Rat caudal vertebrae were frozen in the transverse position with IVDs at the center and stored at -20 °C. AFM was conducted on 8-μm frozen IVD sections using a Bruker Dimension ICON system (Bruker, Billerica, MA, USA). The Young's modulus was measured via AFM. After the appropriate field of view was selected, a 500-1000-MPa probe was used for nanoindentation and to scan the NP tissue. The probe type was an RTESPA-300, the scan rate was 0.8 Hz, and the test mode was quantitative nanomechanical mapping. The scanning range was 2 × 2 μm.

### Isolation of NPCs and TGF-β treatment

The rats were euthanized, and IVDs were obtained. The annulus fibrosus was incised to extract the NP, and this tissue was washed with PBS and cut into small fragments measuring 1.5-2 mm³. The fragments were subsequently digested with 0.2% collagenase II at 37 °C for 3 h. After centrifugation, the supernatant was discarded, and the sediment that had been resuspended in sterile PBS was retained. The sample was evenly seeded into a T25 cell culture flask, and complete DMEM/F12 supplemented with 10% fetal bovine serum (FBS) was added. The cells were cultured in a cell incubator at 37 °C with 5% CO_2_, and the media was changed every three d. NPCs from generations 1‒2 were used throughout the experiments.

TGF-β (10 µg powder) was first centrifuged at 10,000 RPM for 30 s, dissolved to 0.1 mg/ml by adding 100 µl of citric acid, and then diluted to 0.02 mg/ml by adding 400 µl of trehalose solution. TGF-β was added to the nucleus pulposus cells to a TGF-β concentration of 10 nM, and induction was maintained for 7 d, after which the culture medium was exchanged and TGF-β was added again.

### Quantitative PCR

RNAiso Plus (ABclonal, China) was used to extract RNA from the cells. For this, 4 µl of 5 × ABScript III RT Mix and 1 µl of 20 × gDNA Remover Mix (ABclonal, China) were used for reverse transcription into DNA. In each tube, 10 µl of 2 × Universal SYBR Green Fast qPCR Mix (ABclonal, China) was added, along with 1 µl of forward or reverse primer (Sangon Biotech, China), and then, 4 µl of cDNA was added; the final volume was 20 µl. Sterile nuclease-free water was used for qPCR fluorescence quantification. The steps for fluorescence quantification were as follows: predenaturation at 95 °C for 3 min (one cycle), followed by fluorescence signal collection at 95 °C for 5 s and at 60 °C for 30 s for a total of 40 cycles. After the reaction was complete, the amplification curve and melting curve were confirmed via qPCR. The relative mRNA expression levels were determined via the 2^-ΔΔCT^ method.

### Immunofluorescence analysis

NPCs were fixed with 4% paraformaldehyde for 15 min, permeated with 0.5% Triton X-100 (Beyotime, China) for 15 min, and blocked with immunofluorescence blocking solution (Beyotime, China) for 15 min. NPCs were incubated with primary antibodies against COL1 (1:200, Proteintech, China; 67288-1-Ig), FSP-1 (1:200, Proteintech, China; 16105-1-AP), FAP (1:200, Invitrogen, USA; PA5-99313), and FOXO3 (1:200, Proteintech, China; 10849-1-AP) overnight at 4 °C. DAPI was then added, and the mixture was incubated for 5 min in the dark. Finally, the samples were observed under a fluorescence microscope.

### Plasmid construction and transfection

The LAMP2b and MS2 sequences were inserted into the GV712 vector (CMV enhancer-MCS-SV40-puromycin) (Jikai, China) to construct the recombinant plasmid LaM. In addition, the FAP-scFv-LAMP2b-MS2 plasmid (F-LaM) was constructed by inserting FAP-scFvs into the N-terminus of LAMP2b. The G-Mb plasmid was constructed by inserting GFP with MS2bs into the GV127 vector (Jikai, China). The *SKI* mRNA sequence was inserted into G-Mb to construct the GS-Mb plasmid. The plasmids were constructed using a homologous recombination kit. The recombinant plasmids and FAP-scFv sequences were synthesized by the supplier (Jikai, China). The *SKI*-overexpression plasmid (SKI^OE^, CMV enhancer-MCS-3flag-polyA-EF1A-zsGreen-sv40-puromycin) was constructed using the GV657 vector (Jikai, China). Plasmid construction was performed using a homologous recombination kit. The supplier (Jikai, China) provided the sequences and synthesized the plasmids.

Each plasmid was mixed with DMEM/F12 with vortexing in a 1.5-ml EP tube, E-trans was added to the medium, and the mixture was incubated at room temperature for 5-10 min. The suspension was added to cultured HEK-293T cells at a ratio of 1 µg of plasmid/3 µl of transfection reagent/100 µl of serum-free DMEM/F12 medium in 1 ml of medium. After 4-6 h, the status of the cells was observed, and after gently washing with PBS, the medium was replaced with fresh 1:1 DMEM/F12 complete culture medium. The expression of the fluorescently labeled genes on the plasmid was observed 24-48 h post-transfection.

### Exo extraction and identification

HEK-293T cells were cultured in FBS-free DMEM for 2 d. The culture media were subsequently harvested and centrifuged at 500 × g for 10 min and 2000 × g for 30 min to remove dead cells and debris and then 10,000 × g for 1 h to remove large vesicles. The supernatant was collected and ultracentrifuged at 100,000 × g for 70 min at 4 °C using a 70 Ti rotor. The supernatant was carefully removed, and an equal amount of PBS was added to the tube. Finally, 5 µl of PBS was used to collect the Exos, which were stored at -80 °C.

Exos were observed using a G2 Spirit FEI electron transmission microscope (Tecnai, USA). The number and size distribution of the Exos were analyzed via NTA. NTA was performed via a NanoSight NS300 instrument (Malvern, Britain) calibrated with polystyrene microspheres (100 nm). The Exos were characterized by the expression of Exo markers, such as calnexin, TSG101, and CD81. The detailed western blotting procedure is described below.

### Synthesis of pH-responsive hydrogels

#### Synthesis of OSA

Sodium alginate (SA, Aladdin, China) (1 g) was dissolved in 100 ml of distilled water, followed by the addition of 0.7 g of sodium periodate (NaIO4, Aladdin, China). The mixture was stirred in the dark for 48 h. Afterward, 1 ml of glycol was added to the mixture, which was stirred for 30 min. The mixture was transferred to a dialysis bag (2000 Da, MWCO, Millipore) for 3 d of dialysis, after which the solution remaining in the dialysis bag was freeze-dried to obtain OSA, which was characterized by ^1^H NMR (400 MHz, D2O, ppm) and FTIR spectroscopy.

#### Preparation of the dynamic hydrogels

First, 0.5, 1 and 1.5 g of OSA were separately dissolved in 10 ml of PBS to obtain 5, 10 and 15% OSA solutions, respectively. Then, 2 and 3 g of gelatin were dissolved in 10 ml of PBS at 60 °C to prepare 20 and 30% gelatin solutions, respectively. Next, 600 μl of the gelatin mixture was mixed with 200 µl of OSA solution at room temperature to immediately form a complex hydrogel (Gel). Exos (EX) were added. Different combinations of OSA solution and gelatin solution were combined to generate five types of Gel@EX as follows: I, 20% gelatin + 10% OSA; II, 20% gelatin + 15% OSA; III, 30% gelatin + 5% OSA; IV, 30% gelatin + 10% OSA; and V, 30% gelatin + 15% OSA.

### Characterization of the hydrogels

The internal networks of the five types of dynamic hydrogels were observed using a tabletop scanning electron microscope (TM4000 Plus). Exos emitting fluorescence from GFP were encapsulated in the hydrogels and observed by laser scanning confocal fluorescence microscopy (TCS SP8 DIVE). ESEM was used to observe Exo loading.

#### Assessment of compressive properties

Each hydrogel sample was molded into a cylindrical shape with a diameter of 8 mm and a height of 8 mm. A microscale *in situ* mechanical test system (IBTC-300) was used to compress the hydrogel to 30% its original height at a speed of 0.05 mm/s using a 30 N sensor. The compression modulus was calculated as follows:

ε (%) = (Lo-L)/Lo; σ(Pa) = P/A

#### Assessment of tensile properties

The hydrogels from each group were made into cuboids with dimensions of 15 × 5 × 5 mm for the stretching experiments. An IBTC-300 microscale *in situ* mechanical test system was used to stretch the hydrogel at a speed of 0.05 mm/s using a 30 N sensor. The tensile modulus was calculated from the slope of the linear region of the tensile stress-strain curve. The tensile strain and stress were obtained via the following equations:

Ε (%) = (Lo-L)/Lo; σ (Pa) = P/A

#### Assessment of swelling performance

After immersion in 3 ml of PBS at 37 °C for a total of 24 h, the hydrogels from each of the five prepared groups were removed at different time points and weighed on a digital balance. The swelling rate of each hydrogel was calculated as follows:

Swelling rate (%) = (Wt - Wd)/Wd × 100%

#### Assessment of fatigue resistance

The hydrogel samples from each group were processed into cylindrical shapes with a diameter of 8 mm and a height of 8 mm using molds. An IBTC-300 microscale *in situ* mechanical test system was used to compress the hydrogel to 50% of its original height at a speed of 0.05 mm/s using a 30 N sensor for 20 cycles.

### Hydrogel degradation and pH-responsive Exo release experiments

The hydrogels were immersed in PBS (pH 5.5 or 7.0) at 37 °C to evaluate their *in vitro* degradation and Exo release. The hydrogel samples were removed at different time points, and the liquid on the surface of the hydrogel was removed as much as possible. The hydrogel was weighed on a digital balance to calculate its degradation rate. Moreover, the coincubation hydrogel mixture was collected at different time points, and Exo release was determined using a fluorescence spectrophotometer (RF-6000). To assess the *in vivo* degradation of the exosome/hydrogel system, Cy5 was mixed with the exosome/hydrogel and injected into the rat caudal IVD, whereas Cy5 alone was used in the control group. The degradation of the exosome/hydrogel system was observed using a live small animal imaging system (PerkinElmer, USA) after 0, 7, 21 and 28 d.

### Live/dead cell assays

NPCs were added to calcein AM/propidium iodide (PI) detection solution (Beyotime, China). A total of 250 μl of the cell suspension was added to each well of a 24-well plate. The mixture was incubated at 37 °C for 30 min in the dark. After incubation, the sections were observed under a fluorescence microscope (calcein AM emits green fluorescence, Ex/Em = 494/517 nm; PI emits red fluorescence, Ex/Em = 535/617 nm).

### Molecular docking

The crystal structures of SKI (PDB ID: 1SBX) and FOXO3 (PDB ID: 2K86) were obtained from the PDB website. AutoDock4.2 was used for protein preparation, including the addition of H atoms, calculation of the pH environment, protonation or deprotonation of amino acid residues, removal of excess water molecules, and optimization of water molecules and hydrogen bonds. Docking was performed with the HDOCK server, and the first ten conformations out of one hundred output conformations were analyzed to find suitable conformations without limiting the calculations. PyMOL was used for visualization and mapping.

### siRNA transfection

siRNAs targeting rat FOXO3 (rFOXO3A-501: CGAGGAGGACGACGAUGAATT; UUCAUCGUCGUCCUCCUCGTT) and the corresponding scrambled siRNA used as a negative control (NC siRNA) were chemically synthesized by a supplier (Jikai, China). NPCs were evenly spread in 24-well plates until they reached approximately 60% confluence. SiRNA transfection was performed using RNATransMate transfection reagent (Sangon Biotech, China). The siRNA was dissolved in DEPC-treated water, and 1 OD/tube of the siRNA was equivalent to 2.5 nM. Different doses of DEPC were added to water, and then, the siRNA was mixed with the RNATransMate transfection reagent to form solutions with siRNA concentrations of 30, 50, and 100 nM. One microliter of the mixture and 500 µl of serum-free medium were added to each well of a 24-well plate. The cell culture plate was then gently shaken back and forth before incubation at 37 °C for 18-24 h and subsequent detection.

### RNA sequencing

NPCs treated with Gel@EX^ski+scFv^ or Gel@EX^scFv^ plus TGF-β were isolated, and high-throughput RNA sequencing was performed by Majorbio Biotechnology Co., Ltd. (Shanghai, China). Total RNA was extracted from the NPCs on ice via TRIzol. The purity, RNA concentration, and RNA integrity were measured using a NanoDrop 2000 (Thermo Fisher, CA, USA) and an Agilent Bioanalyzer 2100 system (Agilent Technologies, CA, USA). A cDNA library was constructed, and its quality was evaluated. The Illumina NovaSeq platform was used for RNA sequencing. Transcripts were reconstructed using StringTie, and HISAT2 tools software was used to map the clean data to the rat genome. The gene expression levels were evaluated based on fragments per kilobase of transcript per million fragments mapped (FPKM) values. Differentially expressed gene analysis was performed using the DESeq R package (1.10.1). The significantly differentially expressed genes were identified by the corrected *p* value (FDR < 0.05) and |log2foldchange| (|FPKM| ≥ 1). KOBAS software (KOBAS, Surrey, UK) was used to perform the GO and KEGG pathway enrichment analyses.

### Coimmunoprecipitation

To detect protein binding to FOXO3, total cellular protein was extracted by adding precooled RIPA buffer. After the total protein was diluted with PBS to approximately 1 μg/μl, a specific volume of IgG for FOXO3 (1:2000, Proteintech, China, 10849-1-AP) was added to 500 μl of total protein, and the antigen-antibody mixture was slowly shaken overnight at 4 °C. Then, 100 μl of protein A agarose beads (ABclonal, China) were added to capture the antigen-antibody complex, and the antigen-antibody mixture was slowly shaken overnight at 4 °C. The agarose bead-antigen-antibody complex was collected by transient centrifugation at 14,000 rpm for 5 s. The agarose bead-antigen-antibody complex was then mixed with 60 μl of 2 × loading buffer, and the loading sample was boiled for 5 min to free the antigen, antibody, and beads before centrifugation. The supernatant was collected and prepared for western blotting analysis of FOXO3 (1:2000, Proteintech, China, 10849-1-AP) and SKI (1:5000; Santa Cruz, USA; sc-33693).

To detect proteins that bind to SK, the same procedure as that used for SKI IP was used. After IP, the protein was collected and prepared for western blotting analysis of SKI and FOXO3.

### Western blotting

The cells were collected and lysed in RIPA buffer (Epizyme, China), and the protease inhibitor PMSF and other phosphatase inhibitors (Epizyme, China) were added to prevent protein degradation. After the protein concentration was determined, 7.5 or 12.5% colloids were prepared, and electrophoresis was performed (120 V, 90 min). After electrophoresis, the proteins were transferred to a membrane via the wet rotation method (300 mA, 40-90 min), and skim milk powder in TBST was used for blocking. After incubation overnight with a single antibody [COL1 (1:200, Proteintech, China; 67288-1-Ig), FSP-1 (1:10,000, Proteintech, China, 16105-1-AP), FAP (1:2000, Invitrogen, USA, PA5-99313), Calnexin (1:10000, Proteintech, China, 10427-2-AP), TSG101 (1:8000, Proteintech, China, 28283-1-AP), CD81 (1:3000, Proteintech, China, 66866-1-Ig), (1:5000; Santa Cruz, USA; sc-33693), FOXO1 (1:5000, Proteintech, China, 18592-1-AP), FOXO3 (1:2000, Proteintech, China, 10849-1-AP), FOXO4 (1:500, Proteintech, China, 21535-1-AP), FOXO6 (1:500, Proteintech, China, 19122-1-AP), AKT (1:5000, Proteintech, China, 10176-2-AP), p-AKT Ser473 (1:2000, Proteintech, China, 28731-AP), p-AKT Thr342 (1:500, Invitrogen, USA, PA5-95669), p-FOXO3 Ser253 (1:500, Abcam, Britain, ab154786), and β-ACTIN (1:50,000, Proteintech, China)], the membranes were washed three times with TBST, and the secondary antibody, labeled with HRP and goat anti-mouse/rabbit IgG (1:500, Yamei, China, LF101/102), was added for incubation at room temperature for 1 h. Then, the membranes were washed with TBST three times before development and imaging (Bio-Rad, USA).

### Statistical analysis

The data are presented as the means ± standard deviations. All experiments were performed independently with at least three replicates. The normality of the data distribution was tested via the Shapiro‒Wilk test, and the difference between two groups was evaluated via the t test or Wilcoxon rank sum test. For multiple group comparisons, one-way or two-way ANOVA was used. All statistical analyses were performed using SPSS software version 26.0 (IL, USA). P value < 0.05 was considered to indicate statistical significance.

## Supplementary Material

Supplementary figures and tables.

## Abbreviations

NP: nucleus pulposus
IVDD: intervertebral disc degeneration
NPC: nucleus pulposus cell
OSA: oxidized sodium alginate
ECM: extracellular matrix
IVD: intervertebral disc
SKI: Cellular Sloan Kettering Institute
Exo: exosomes
COL1: collagen type I
FAP: fibroblast activation protein-α
FSP-1: fibroblast protein-1
scFv: single-chain variable fragments
scRNA-seq: single-cell RNA sequencing
KEGG: Kyoto Encyclopedia of Genes and Genomes
GO: Gene Ontology
CC: cellular component
MF: molecular function
GSEA: gene set enrichment analysis
HE: hematoxylin and eosin
SO-FO: safranin O-fast green
AFM: atomic force microscopy
SKI^OE^: SKI overexpression plasmid
GFP: green fluorescent protein
NTA: nanoparticle tracking analysis
TEM: transmission electron microscopy
NMR: nuclear magnetic resonance
FTIR: Fourier transform infrared
ESEM: environmental scanning electron microscopy
DEG: differentially expressed gene
NIH: National Institutes of Health
FBS: fetal bovine serum
siRNA: small interfering RNA
