# Supplementary material for: An anti-FAP-scFv-functionalized exosome-carrying hydrogel delivers SKI mRNA to fibrotic nucleus pulposus cells to alleviate intervertebral disc degeneration by regulating FOXO3
Source: Theranostics. 2025 Mar 3;15(9):3877–99. doi: 10.7150/thno.107776 (PMC11980650; doi:10.7150/thno.107776)
Supplement: Supplementary file 1 — Supplementary figures and tables. [file thnov15p3877s1.pdf]

Supporting Information

An anti-FAP-scFv-functionalized exosome-carrying hydrogel delivers *SKI* mRNA to fibrotic nucleus pulposus cells to alleviate intervertebral disc degeneration by regulating FOXO3

Yueyang Li[1,2,3#], Yu Zhai[1,2,3][#], Tianling Wang[1,2,3], Siya Wang[4], Yu Tian[5], Zhiqun Bian[1,2,3], Yang Zhang[1,2,3], Chao Zhang[1,2,3]*, Minghan Liu[1,2,3]*, Changqing Li[1,2,3]**

Table S1. Hematological parameter at 8 w after injection 10 μl exosome/hydrogel.

n = 6, SD male rat							
	Sham	TGF- β	TGF-β+Gel	TGF- β +Gel@EX	TGF- β +Gel@EX$^{s}_{cFv}$	TGF- β +Gel@EX$^{s}_{ki}$	TGF- β+Gel@EX$_{ski+scFv}$
	Mean±SD	Mean±SD	Mean±SD	Mean±SD	Mean±SD	Mean±SD	Mean±SD
CRP (mg/L)	0.42±0.24	0.31±0.16	1.21±0.27	0.83±0.42	0.92±0.43	0.62±0.26	0.58±0.31
WBC (× 10^9/L)	8.74±0.22	7.32±0.56	8.12±1.25	7.82±2.13	7.73±1.32	8.19±0.32	8.52±0.44
NEUT (%)	14.11±0.54	13.86±0.25	15.11±1.14	12.75±2.43	13.43±1.04	14.19±0.93	16.38±1.03
LY (%)	74.23±3.16	76.73±4.32	70.73±3.76	74.43±2.52	72.32±2.19	74.27±2.31	77.54±3.29
MONO (%)	3.53±0.30	4.71±0.26	5.11±0.12	4.42±1.31	4.36±2.17	3.28±0.73	4.01±0.62
EO (%)	0.11±0.04	0.18±0.02	0.08±0.04	0.03±0.01	0.04±0.02	0.11±0.05	0.19±0.09
HGB (g/L)	155±4.42	161±7.16	156±6.27	172±8.67	164±4.74	148±2.17	151±3.93
RBC (× 10^{12}/L)	7.52±0.42	7.27±0.38	7.35±0.26	7.81±1.46	7.24±1.07	7.12±0.36	7.73±0.42
HCT (%)	42.31±1.80	41.16±2.32	46.16±5.49	43.63±4.29	49.32±5.01	44.37±1.34	41.36±2.03
MCV (fL)	57.53±1.64	54.34±1.64	59.82±1.54	51.28±3.28	53.52±1.98	51.78±4.28	54.19±2.29
MCH (pg)	20.13±1.19	21.23±1.19	22.18±1.32	20.37±2.17	23.64±3.96	20.47±2.43	18.93±1.32
MCHC (g/L)	321±12.17	319±10.24	337±11.23	328±9.17	347±4.63	348±14.35	329±11.73
RDW-CV (%)	13.21±0.62	14.17±0.45	12.71±1.29	15.27±2.12	14.45±3.67	15.23±3.34	13.48±1.08
RDW-SD (fL)	27.22±3.21	28.14±2.17	23.27±3.28	24.36±2.37	25.16±4.93	24.17±6.58	26.32±3.81
PLT (× 10^9/L)	863±41.21	845±36.12	869±32.37	832±28.17	843±21.29	856±42.47	890±48.12
PCT (%)	0.32±0.17	0.25±0.16	0.28±0.09	0.25±0.18	0.30±0.19	0.44±0.27	0.58±0.23
MPV (fL)	7.4±0.24	6.4±0.17	6.1±0.26	7.4±0.46	7.8±0.89	7.3±2.18	6.3±1.36
PDW (%)	16.31±1.42	17.14±1.26	14.29±1.37	15.43±2.74	16.38±4.23	16.21±1.18	14.68±2.39

CRP: c-reactive protein; WBC: white blood cell; NEUT: neutrophils; LY: lymphocytes; MONO: monocytes; EO: eosinophils; HGB: hemoglobin; RBC: red blood cell; HCT: hematocrit; MCV: mean corpuscular volume; MCH: mean corpuscular hemoglobin; MCHC: mean corpuscular hemoglobin concentration; RDW-CV: red cell

distribution width coefficient of variation; RDW-SD: red cell distribution width-standard deviation; PLT: platelet; PCT: platelet crit; MPV: mean platelet volume; PDW: platelet distribution width

Table S2. Blood chemistry parameter at 8 w after injection 10 μl exosome/hydrogel.

	n = 6, SD male rat						
	Sham	TGF-β	TGF-β+Gel	TGF-β +Gel@EX	TGF-β +Gel@EXscFv	TGF-β +Gel@EXski	TGF-β+Gel@EXski+scFv
	Mean±SD	Mean±SD	Mean±SD	Mean±SD	Mean±SD	Mean±SD	Mean±SD
ALT (U/L)	31.78±2.57	29.3±3.36	28.52±1.47	34.47±3.77	29.56±3.34	25.19±2.55	27.95±3.9
AST (U/L)	80.47±4.38	82.65±4.13	84.92±1.93	80.87±3.51	80.22±4.6	84.46±6.02	82.62±7.69
ALP (U/L)	227±31.04	248±39.39	285±31.44	268±31.47	288±32.03	225±33.22	254±30.94
GGT (U/L)	0.82±0.2	0.97±0.16	0.83±0.28	0.78±0.23	0.8±0.23	0.6±0.1	0.51±0.21
Glu (mg/dL)	143±12.62	149±14.3	150±15.39	147±14.43	133±18.23	139±14.61	142±17.87
BUN (mg/dL)	11.63±1.56	13.5±3.51	14.54±4.5	13.67±3.96	13.05±1.37	10.08±1.85	10.87±2.43
Crea (mg/dL)	0.61±0.23	0.77±0.46	0.78±0.4	0.79±0.45	0.46±0.4	0.42±0.29	0.43±0.41
T-Bili (mg/dL)	0.02±0.02	0.03±0.02	0.03±0.02	0.03±0.01	0.02±0.01	0.04±0.01	0.05±0.02
T-Chol (mg/dL)	84±2	80±4	78±1	80±5	80±1	82±3	77±3
TG (mg/dL)	55±2	52±13	53±6	54±11	58±13	52±8	50±2
TP (g/dL)	7.04±1.36	5.08±1.61	6.98±1.14	6.14±1.94	4.28±1.23	4.38±1.46	7.11±1.28
Alb (g/dL)	2.27±1.45	3.5±1.4	3.39±1.34	3.84±1.78	2.23±1.62	3.38±1.09	2.12±1.2
P (mg/dL)	5.47±1.04	6.44±2.95	6.39±2.39	6.35±1.78	6.03±2.42	5.27±1.25	5.15±1.67
Ca (mg/dL)	11.87±1.4	11.79±2.73	11.68±1.58	11.96±2.45	11.94±2.79	12.24±2.21	11.82±1.98
Na (mmol/L)	149±1.22	149±2.57	149±2.32	146±1.69	146±2.29	150±2.5	149±1.4
K (mmol/L)	4.6±0.35	4.6±0.5	4.6±0.34	4.5±0.39	4.5±0.3	4.5±0.42	4.6±0.48
Cl (mmol/L)	110±6	110±9	113±10	119±10	117±8	120±9	114±6
ALT (U/L)	31.6±4.44	30.55±2.43	30.92±3.35	30.38±4.42	31.78±4.8	31.18±2.18	31.95±2.37
AST (U/L)	70.14±3.59	81.55±5.75	73.2±5.1	76.88±1.05	71.85±3.41	78.14±7.72	84.82±4.46
ALP (U/L)	277±42.14	265±48.15	255±40.79	252±43.97	296±43.12	295±42.15	268±40.34
GGT (U/L)	0.34±0.29	0.98±0.27	0.67±0.28	0.4±0.21	0.89±0.11	0.89±0.19	0.33±0.25
Glu (mg/dL)	151±15.81	145±20.61	152±16.81	154±19.92	151±20.67	149±19.47	153±17.85

ALT, alanine aminotransferase; AST, aspartate aminotransferase; ALP, alkaline phosphatase; GGT, gamma-glutamyl transpeptidase; GLU, glucose; BUN, blood urea nitrogen; Crea, creatinine; T-Bili, total bilirubin; T-Chol, total chloesterol; TG, triglyceride; TP, total protein; Alb, albumin; P, phosphorus, Ca, calcium; Na, sodium; K, potassium; Cl, Chlorde.

Table S3. Hematological parameter at 4 w after subcutaneous implantation 10 μl exosome/hydrogel.

n = 6, SD male rat						
	Sham	Gel	Gel@EX	Gel@EX^{scFv}	Gel@EX^{ski}	Gel@EX$^{ski+scFv}$
	Mean±SD	Mean±SD	Mean±SD	Mean±SD	Mean±SD	Mean±SD
CRP (mg/L)	0.36±0.15	1.37±1.12	0.81±0.19	0.91±0.44	0.6±0.14	0.64±0.25
WBC (× 10^9/L)	7.23±0.16	8.31±2.13	7.6±0.92	7.01±0.74	7.27±0.39	7.48±0.38
NEUT (%)	15.12±0.33	14.32±2.17	12.42±1.97	12.34±1.66	13.33±2.6	13.86±2.79
LY (%)	74.33±4.21	72.43±2.37	75.67±3.65	74.23±3.52	75.13±2.34	74.1±2.21
MONO (%)	3.28±0.14	4.34±1.37	3.62±1.3	4.57±1.36	3.53±1.89	4.72±1.76
EO (%)	0.09±0.02	0.05±0.02	0.05±0.03	0.06±0.02	0.03±0.01	0.03±0.02
HGB (g/L)	162±5.11	161±5.37	170±2.64	166±2.89	165±2.58	163±3.44
RBC (× 10^{12}/L)	7.89±1.22	7.71±1.23	7.14±1.71	6.59±0.14	7.42±1.03	7.96±1.97
HCT (%)	43.13±2.20	45.37±4.74	46.43±3.75	47.19±3.41	46.01±1.17	49.76±2.61
MCV (fL)	58.12±3.21	57.32±2.18	53.05±2.14	50.63±2.5	51.33±3.75	51.73±1.16
MCH (pg)	21.26±2.28	21.43±2.37	22.9±2.01	23.03±3.29	22.09±1.29	21.97±4.2
MCHC (g/L)	313±11.57	327±12.37	325±8.29	343±8.87	347±10.68	326±8.5
RDW-CV (%)	13.21±0.62	11.57±2.18	14.66±2.82	10.44±2.54	10.81±4.5	13.93±1.4
RDW-SD (fL)	25.27±2.69	24.37±1.23	24.36±2.37	25.16±4.93	24.17±6.58	26.32±3.81
PLT (× 10^9/L)	852±42.16	837±31.27	822±37.15	849±42.31	829±43.12	808±37.93
PCT (%)	0.14±0.05	0.44±0.31	0.38±0.12	0.27±0.18	0.48±0.12	0.31±0.11
MPV (fL)	7.3±0.38	6.6±0.38	6.94±1.01	6.44±2.3	6.33±1.65	6.05±1.58
PDW (%)	15.12±2.17	16.21±2.37	14.88±1.56	14.3±1.44	14.45±1.58	15.94±2.98

CRP: c-reactive protein; WBC: white blood cell; NEUT: neutrophils; LY: lymphocytes; MONO: monocytes; EO: eosinophils; HGB: hemoglobin; RBC: red blood cell; HCT: hematocrit; MCV: mean corpuscular volume; MCH: mean corpuscular hemoglobin; MCHC: mean corpuscular hemoglobin concentration; RDW-CV: red cell distribution width coefficient of variation; RDW-SD: red cell distribution width-standard deviation; PLT: platelet; PCT: platelet crit; MPV: mean platelet volume; PDW: platelet distribution width

Table S4. Blood chemistry parameter at 4 w after subcutaneous implantation 10 µl exosome/hydrogel.

n = 6, SD male rat						
	Sham	Gel	Gel@EX	Gel@EX^{scFv}	Gel@EX^{ski}	Gel@EX$^{ski+scFv}$
	Mean±SD	Mean ± SD	Mean ± SD	Mean ± SD	Mean ± SD	Mean±SD
ALT (U/L)	25.8±4.67	28.21±4.79	34.34±1.58	26.72±2.78	26.79±3.97	33.39±3.19
AST (U/L)	84.58±3.62	75.88±3.91	81.14±1.62	77.35±1.75	83.23±2.78	77.84±2.19
ALP (U/L)	259±34.39	256±28.75	256±27.36	237±32.86	267±26.1	255±27.4
GGT (U/L)	0.61±0.17	0.62±0.17	0.76±0.15	0.68±0.3	0.73±0.16	0.77±0.23
Glu (mg/dL)	143±5.79	145±5.59	146±5.19	137±8.33	140±8.55	146±7.28
BUN (mg/dL)	11.91±3.33	14.69±2.03	13.49±2.26	13.25±2.4	10.52±2.38	11.04±3.24
Crea (mg/dL)	1.13±0.18	1.36±0.46	1.25±0.19	0.57±0.48	0.69±0.32	1.47±0.42
T-Bili (mg/dL)	0.02±0.01	0.04±0.01	0.02±0.01	0.04±0.01	0.05±0.02	0.06±0.03
T-Chol (mg/dL)	81±5	79±1	82±5	83±5	79±5	79±4
TG (mg/dL)	46±3	54±2	45±4	54±1	51±3	50±2
TP (g/dL)	4.48±3.94	5.04±4.65	6.74±2.9	5.03±3.94	6.47±2.91	6.55±4.76
Alb (g/dL)	1.2±1.32	1.01±1.5	2.74±1.12	2.24±1.28	1.06±1.38	1.47±0.63
P (mg/dL)	4.32±0.99	5.58±0.93	6.58±0.85	5.21±0.8	4.91±1.01	4.25±0.72
Ca (mg/dL)	10.71±1.07	7.81±0.99	9.63±1.34	9.56±1.05	9.84±1.21	7.33±0.8
Na (mmol/L)	145±0.91	147±1.4	149±0.87	146±0.52	149±1.19	146±1.46
K (mmol/L)	4.5±0.23	4.6±0.23	4.5±0.25	4.5±0.23	4.6±0.25	4.5±0.16
Cl (mmol/L)	114±3	120±4	111±2	115±2	117±1	113±3
ALT (U/L)	28.52±2.09	30.75±1.25	30.57±1.11	34.21±2.72	32.31±3.33	33.73±3.26
AST (U/L)	62.1±5.61	77.84±3.96	69.07±5.22	63.62±4.27	66.46±6.41	69.05±5.25
ALP (U/L)	223±36.91	266±39.62	224±33.02	244±39.12	274±38.35	278±36.95
GGT (U/L)	0.23±0.18	0.39±0.19	0.34±0.11	0.46±0.19	0.4±0.29	0.45±0.27
Glu (mg/dL)	153±4.54	155±3.74	140±4.55	156±4.48	146±4.07	140±3.6

ALT, alanine aminotransferase; AST, aspartate aminotransferase; ALP, alkaline phosphatase; GGT, gamma-glutamyl transpeptidase; GLU, glucose; BUN, blood urea nitrogen; Crea, creatinine; T-Bili, total bilirubin; T-Chol, total chloesterol; TG, triglyceride; TP, total protein; Alb, albumin; P, phosphorus, Ca, calcium; Na, sodium; K, potassium; Cl, Chlorde.

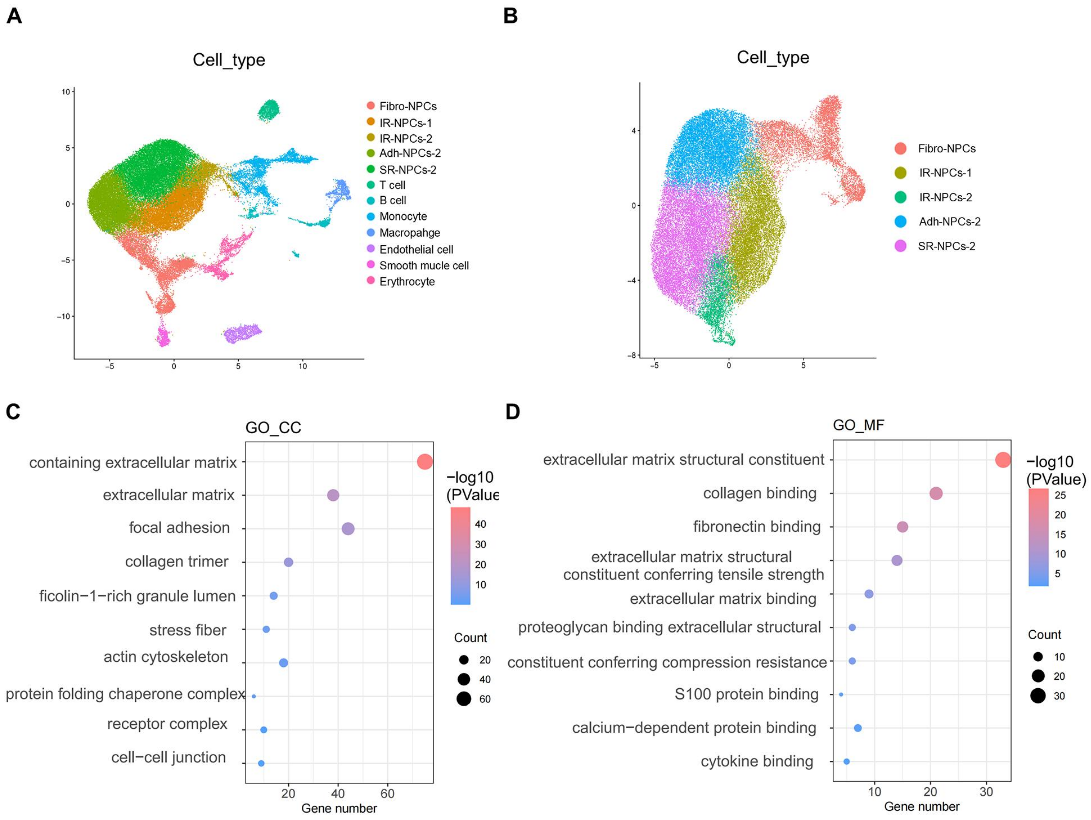

Figure S1: Results of scRNA-seq analysis of the GEO database (GSE244889). **(A, B)** Schematic UMAP of the total cells in the NP and subsets of NPCs with a resolution of 0.5. **(C, D)** GO_CC and GO_MF analysis of the GEO database.

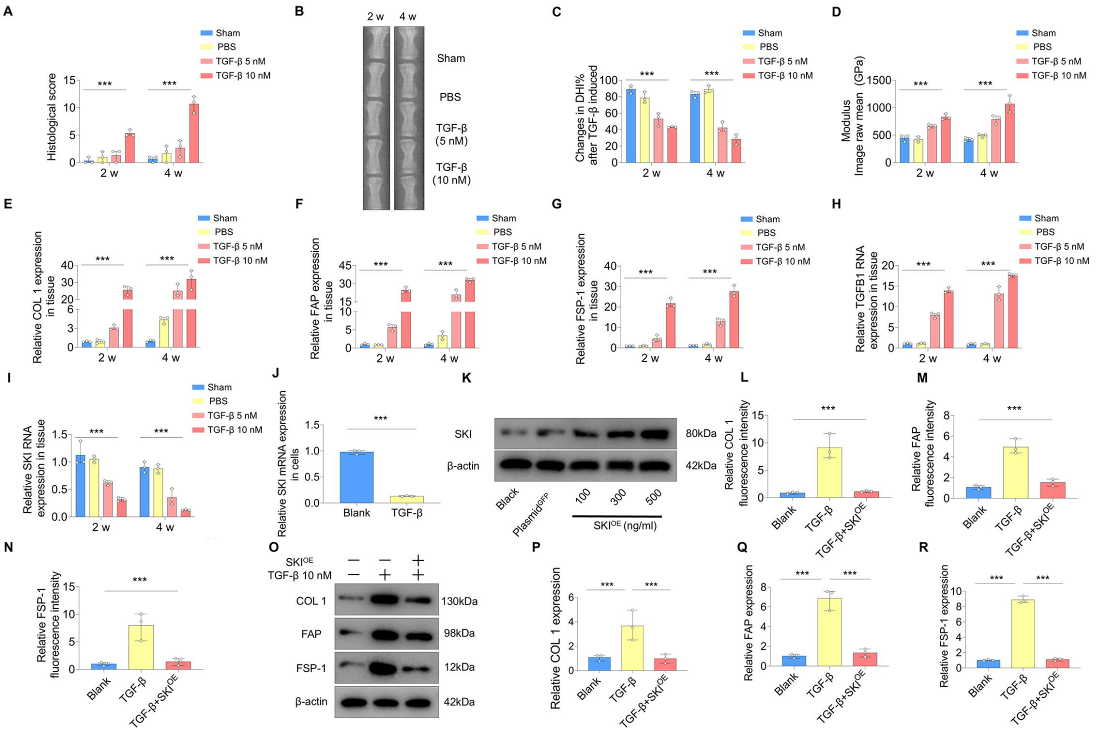

Figure S2: TGF-β-induced NP fibrosis model construction and validation in *vitro* and in *vivo*. **(A)** Histological scores from HE staining at 2 and 4 w Sham gourp or after injection with 5 μl of PBS or 5 nM or 10 nM TGF-β via a microsyringes, n = 3, statistical differences were determined by two-way ANOVA, ***P < 0.001. **(B, C)** X ray and statistical analysis of the DHI% changes at 2 and 4 w Sham gourp or after injection with 5 μl of PBS or 5 nM or 10 nM TGF-β via a microsyringes, statistical differences were determined by two-way ANOVA, ***P < 0.001. **(D)** Statistical analysis of the Young's modulus of intervertebral disc sections by AFM at 2 and 4 w Sham group or after injection with 5 μl of PBS or 5 nM or 10 nM TGF-β via a microsyringes, n = 3, statistical differences were determined by two-way ANOVA, ***P < 0.001. **(E-G)** Western blot analysis of COL1, FAP and FSP-1 in NP at 2 and 4 w Sham group or after injection with 5 μl of PBS or 5 nM or 10 nM TGF-β via a microsyringes, n = 3, statistical differences were determined by two-way ANOVA, ***P < 0.001. **(H, I)** PCR analysis of *TGFB1* and *SKI* at 2 and 4 w Sham group or after injection with 5 μl of PBS or 5 or 10 nM TGF-β via a microsyringes, n = 3, statistical differences were determined by two-way ANOVA, ***P < 0.001. **(J)** PCR analysis of *SKI* of NPCs after induction with TGF-β (10 nM), n = 3, statistical differences were determined by Student's t tests, ***P < 0.001. **(K)** Western blot

analysis of SKI expression after SKI overexpression induction. **(L-N)** Statistical analysis of the immunofluorescence of COL1, FAP and FSP-1 expression in NPCs induced by TGF-β (10 nM) and SKI^{OE}, n = 3, statistical differences were determined by one-way ANOVA, ***P < 0.001. **(O-R)** Western blot analysis and statistical analysis of COL1, FAP and FSP-1 expression in NPCs with *SKI* overexpression after TGF-β (10 nM) induction, n = 3, statistical differences were determined by one-way ANOVA, ***P < 0.001.

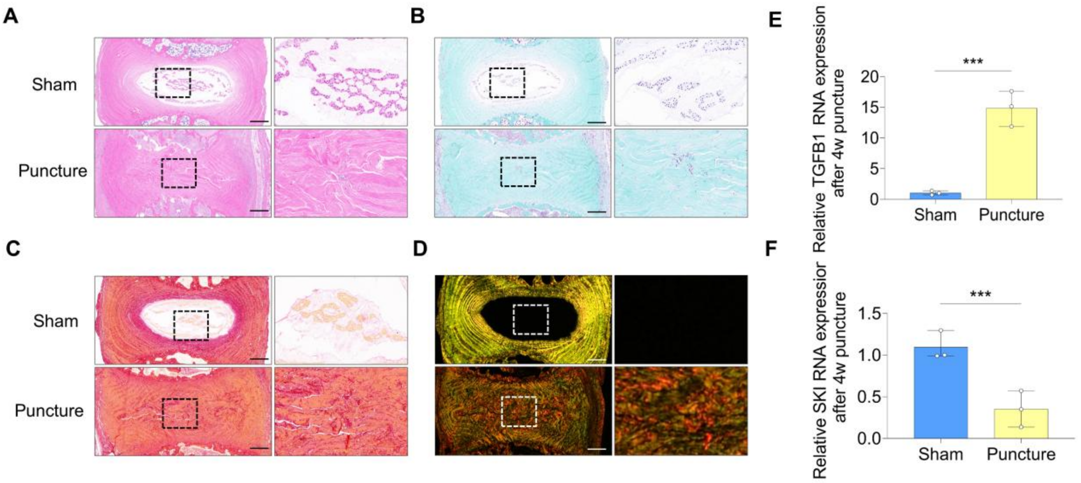

Figure S3: Validation of the NP fibrosis in a PIDD-induced NP fibrosis model. **(A)** HE staining evaluation of the PIDD model at 4 w; scale bar = 200 μm, n = 3. **(B)** SO-FO staining evaluation of the PIDD model at 4 w; scale bar = 200 μm, n = 3. **(C, D)** White light and polarized light sirius red staining evaluation of the thickness of IVD fibers in the PIDD model at 4 w; scale bar = 200 μm, n = 3. **(E, F)** PCR analysis of *TGFB1* and *SKI* expression in the PIDD model at 4 w, n = 3, statistical differences were determined by Student's t tests, ***P < 0.001.

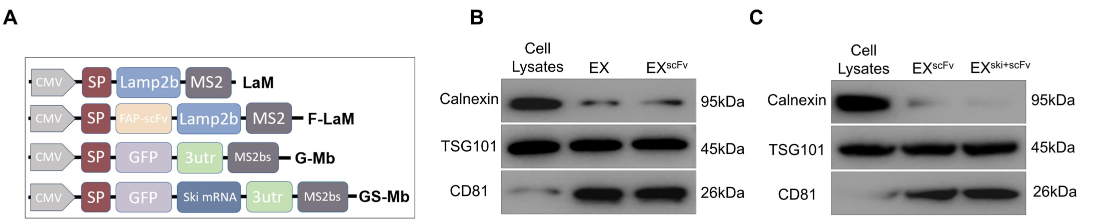

Figure S4: Functionalized Exo construction and synthesis. **(A)** Schematic representation of the construction of the Lam, F-Lam, G-Mb, and GS-Mb plasmids. **(B, C)** Western blot analysis of Calnexin, TSG101, and CD81 protein expression in EX, EX^{scFv}, EX^{ski}, and EX$^{ski+scFv}$, n = 3.

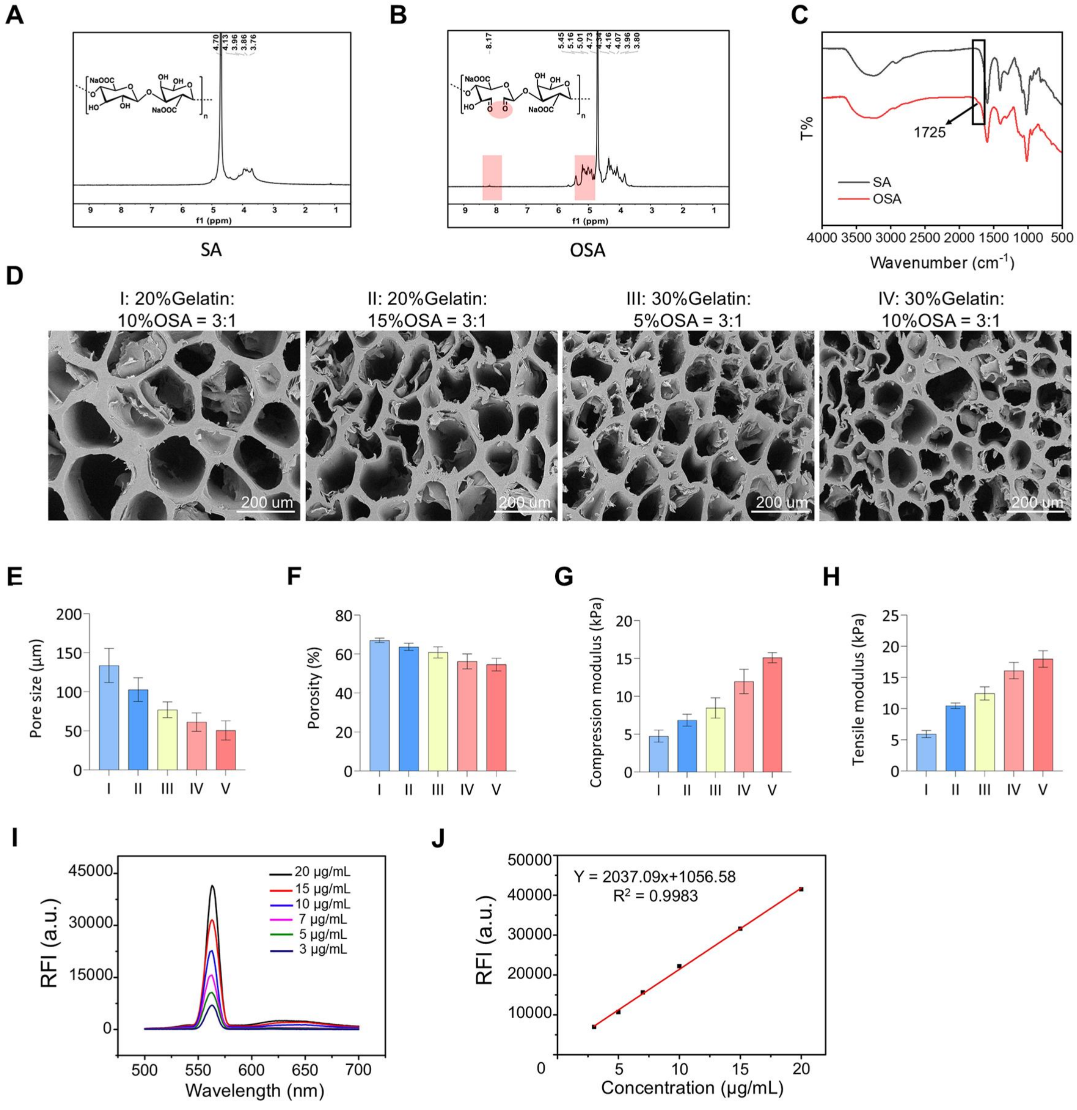

Figure S5: Identification of Gel@EX$^{ski+scFv}$. **(A, B)** Hydrogel magnetic resonance spectroscopy verified that new peaks appeared in OSA compared with SA, indicating the formation of aldehyde groups. **(C)** FTIR spectroscopic analysis of the

hydrogel revealed that the characteristic absorption peak of the aldehyde group appeared at 1725 cm-1, confirming the successful introduction of the aldehyde group on the OSA chain. **(D)** Scanning electron microscopy of hydrogels from I-IV, n = 3. **(E, F)** Pore size and porosity of hydrogels from I-V, n = 3. **(G, H)** Compression and tensile modulus of hydrogels from I-V, n = 3. **(I, J)** Fluorospectrophotometer analysis of Gel@EX$^{ski+scFv}$ and calculation of the standard curve revealed that the wavelength of the Exos was approximately 570 nm.

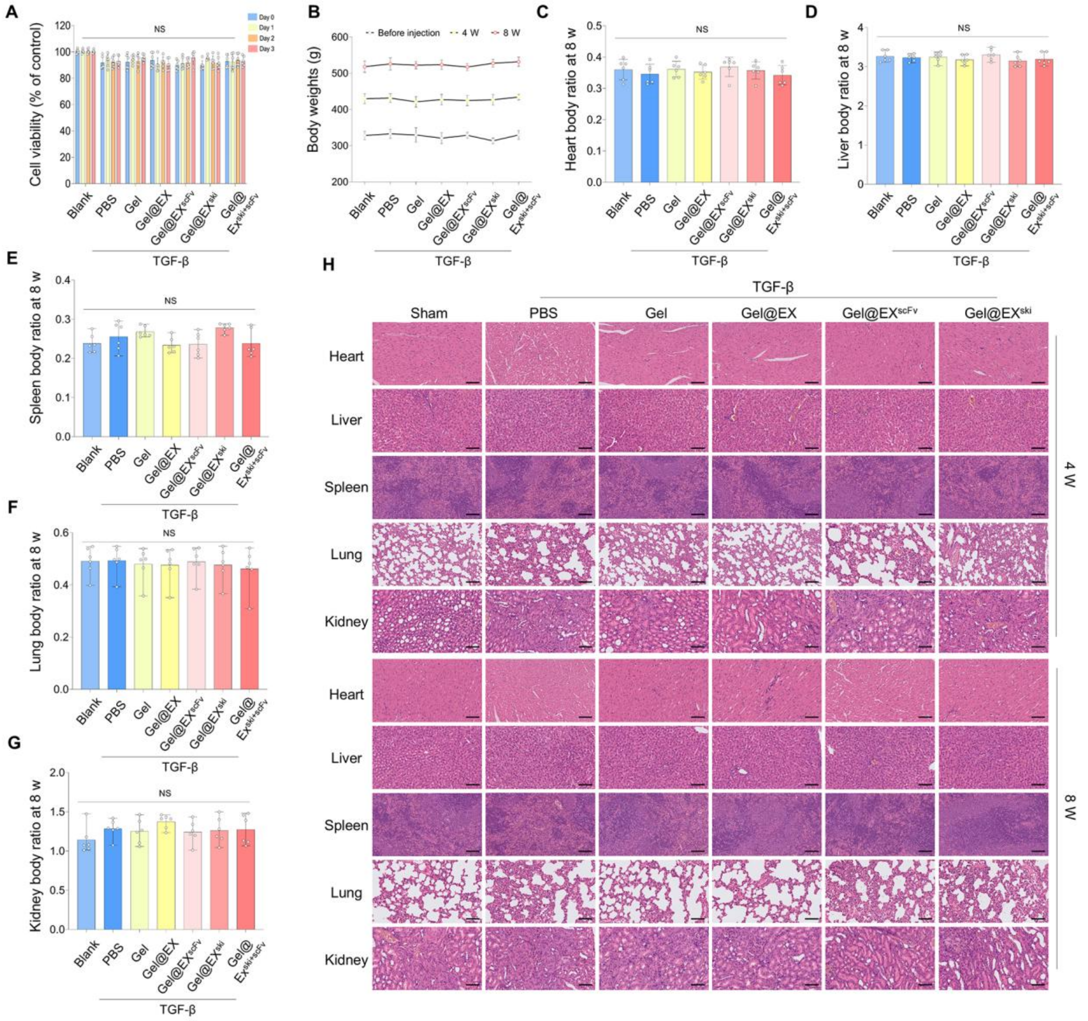

Figure S6: Validation of biocompatibility of Gel@EX$^{ski+scFv}$. **(A)** Statistical analysis of CCK8 of fibrotic NPCs after 7 d of TGF-β-induced and coculture with different exosome/hydrogel system, n = 6, statistical differences were determined by one-way ANOVA, NS: no significance. **(B)** Body weights of rats before injection, 4 w and 8 w after injection of 10 μl different exosome/hydrogel system after TGF-β-induced NP fibrosis, n = 6. **(C-G)** Statistical analysis of organ weight ratio at 8 w after

injection of 10 µl different exosome/hydrogel system after TGF-β-induced NP fibrosis, n = 6, statistical differences were determined by one-way ANOVA, NS: no significance. **(H)** HE staining evaluation of the organs of rats after 4 w and 8 w injection of 10 µl different exosome/hydrogel system after TGF-β-induced NP fibrosis, Scale bar = 50 µm, n = 6.

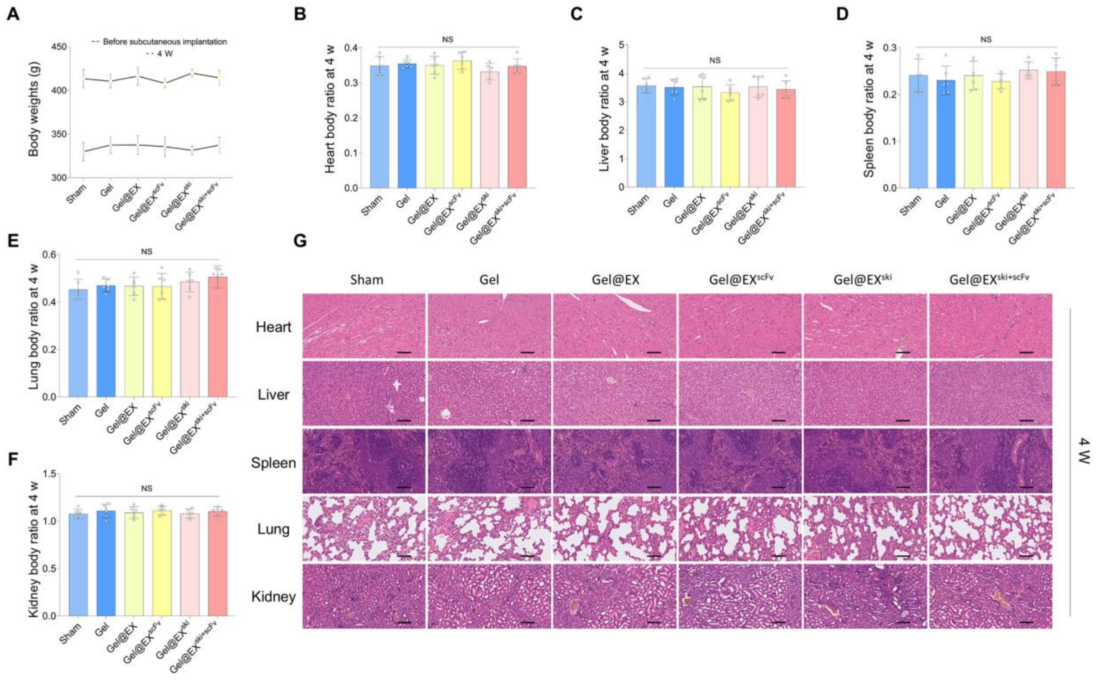

Figure S7: Validation of biocompatibility of Gel@EX$^{ski+scFv}$. **(A)** Body weights of rats before subcutaneous implantation and 4 w subcutaneous implantation of 10 µl different exosome/hydrogel system, n = 6. **(B-F)** Statistical analysis of organ weight ratio at 4 w after subcutaneous implantation of 10 µl different exosome/hydrogel system, n = 6, statistical differences were determined by one-way ANOVA, NS: no significance. **(G)** HE staining evaluation of the organs of rats after 4 w subcutaneous implantation of 10 µl different exosome/hydrogel system, scale bar = 50 µm, n = 6.

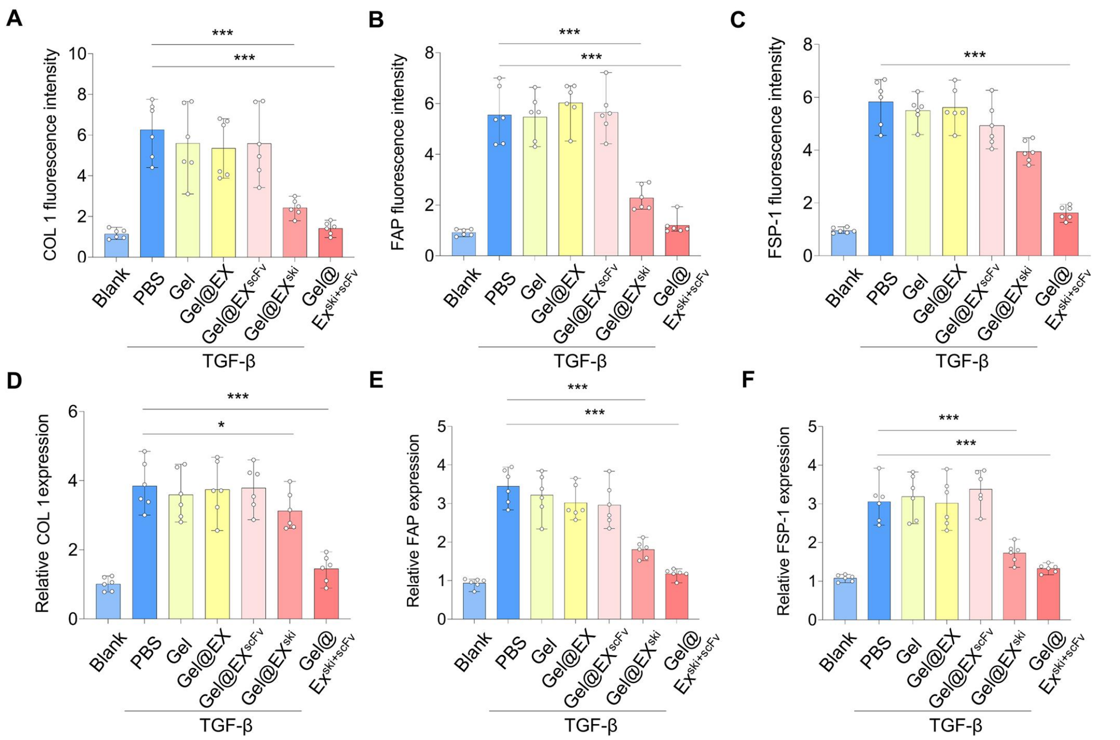

Figure S8: Verification of the *in vitro* therapeutic effect of Gel@EX$^{ski+scFv}$. **(A-C)** Statistical analysis of the immunofluorescence of COL1, FAP and FSP-1 expression in NPCs induced by TGF-β and different exosome/hydrogel system, n = 6, statistical differences were determined by one-way ANOVA, ***P < 0.001. **(D-F)** Statistical analysis of the western blot of COL1, FAP and FSP-1 expression in NPCs induced by TGF-β and different exosome/hydrogel system, n = 6, statistical differences were determined by one-way ANOVA, ***P < 0.001, *P < 0.05.

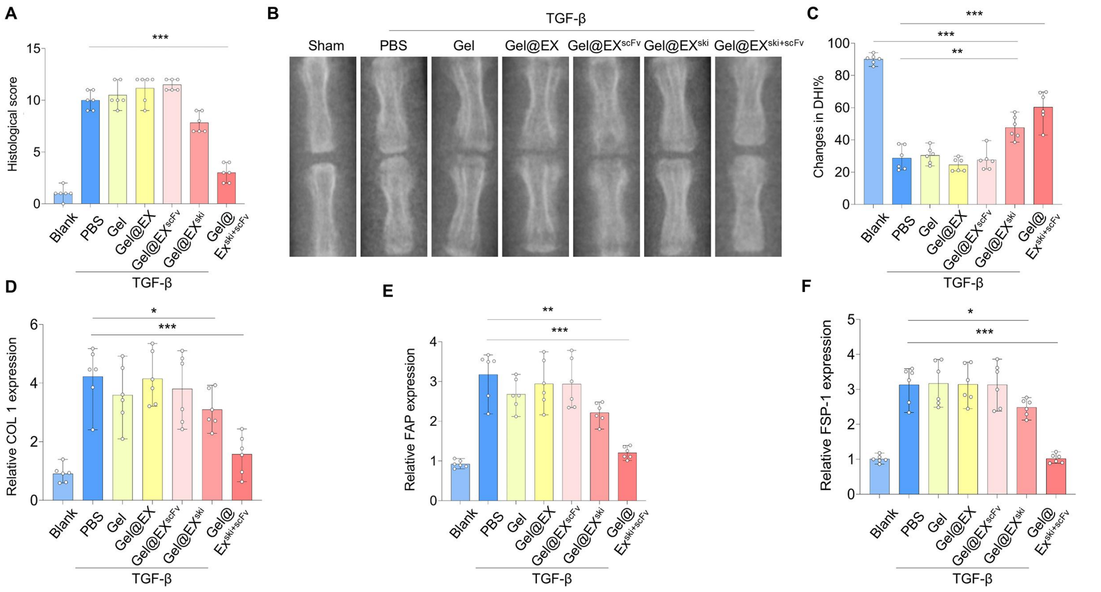

Figure S9: Verification of the *in vivo* therapeutic effect of Gel@EX$^{ski+scFv}$. **(A)** histological scores after injection 10 µl with different exosome/hydrogel systems after TGF-β-induced NP fibrosis, n = 6, statistical differences were determined by one-way ANOVA, ***P < 0.001. **(B, C)** X ray and DHI% changes after injection 10 µl with different exosome/hydrogel systems after TGF-β-induced NP fibrosis, n = 6, statistical differences were determined by one-way ANOVA, ***P < 0.001, **P< 0.01. **(D-F)** Western blot analysis of COL1, FAP and FSP-1 expression after injection 10 µl with different exosome/hydrogel systems after TGF-β-induced NP fibrosis; n = 6, statistical differences were determined by one-way ANOVA, ***P < 0.001, **P < 0.01, *P< 0.05.

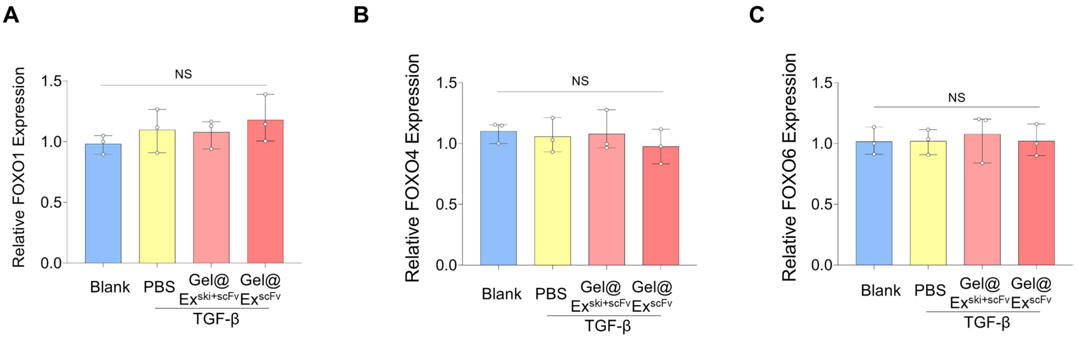

Figure S10: Verification of the FOXO proteins level regulated by SKI. **(A-C)** Western blot analysis of FOXO1, FOXO4, and FOXO6 expression in the FOXO family under TGF-β induction and after SKI overexpression, n = 3, statistical differences were determined by one-way ANOVA, NS: no significance.

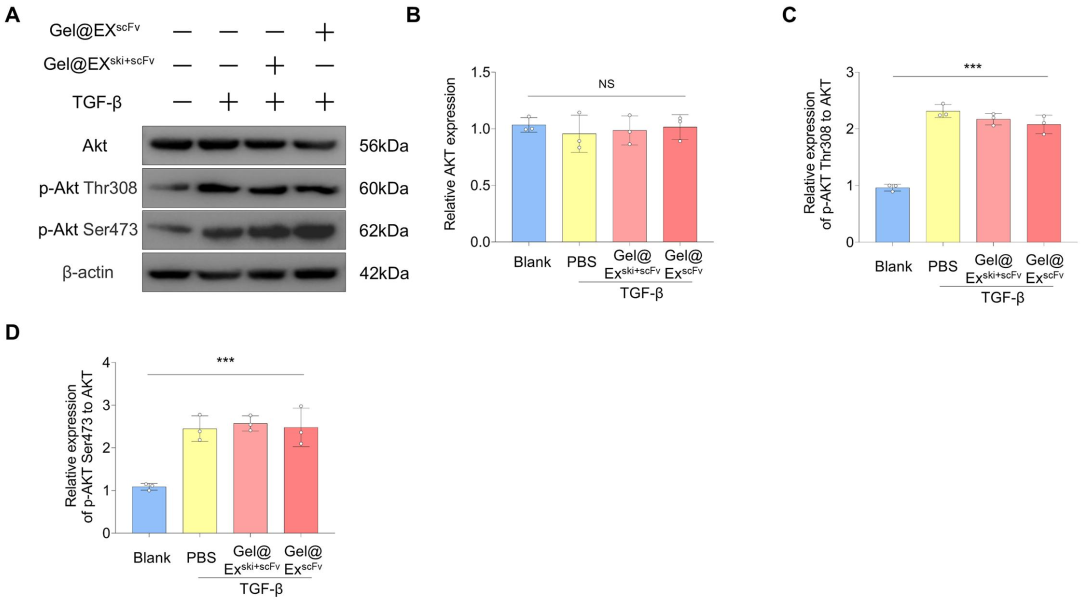

Figure S11: Verification of the mechanism of SKI in regulating TGF-β signaling pathway. **(A, B)** Western blot analysis of AKT expression under TGF-β induction and after *SKI* overexpression, n = 3, statistical differences were determined by one-way ANOVA, NS: no significance. **(C, D)** Western blot analysis of p-AKT Thr308 and p-AKT Ser473 expression in response to AKT, n = 3, statistical differences were determined by one-way ANOVA, ***P < 0.001.
